# Chemo-photodynamic therapy with light-triggered disassembly of theranostic nanoplatform in combination with checkpoint blockade for immunotherapy of hepatocellular carcinoma

**DOI:** 10.1186/s12951-021-01101-1

**Published:** 2021-10-30

**Authors:** Jianjun Xu, Qichang Zheng, Xiang Cheng, Shaobo Hu, Chen Zhang, Xing Zhou, Ping Sun, Weimin Wang, Zhe Su, Tianhao Zou, Zifang Song, Yun Xia, Xiaoqing Yi, Yang Gao

**Affiliations:** 1 Department of Hepatobiliary Surgery, Union Hospital, Tongji Medical College, Huazhong University of Science and Technology, Wuhan, 430022 China; 2 Department of General Surgery, Tongji Hospital, Tongji Medical College, Huazhong University of Science and Technology, Wuhan, 430030 China; 3 Key Laboratory of Prevention and Treatment of Cardiovascular and Cerebrovascular Diseases, Ministry of Education, College of Pharmacy, Key Laboratory of Biomaterials and Biofabrication in Tissue Engineering of Jiangxi Province, Gannan Medical University, Ganzhou, 341000 China

**Keywords:** Theranostic nanoplatform, Aggregation-induced emission (AIE), Light-triggered nanomedicine, Cancer immunotherapy, Immune checkpoint blockade, Photodynamic therapy, Chemotherapy

## Abstract

**Background:**

Hepatocellular carcinoma (HCC) is a common malignant tumor with high rate of metastasis and recurrence. Although immune checkpoint blockade (ICB) has emerged as a promising type of immunotherapy in advanced HCC, treatment with ICB alone achieves an objective remission rate less than 20%. Thus, combination therapy strategies is needed to improve the treatment response rate and therapeutic effect.

**Methods:**

A light-triggered disassembly of nanoplatform (TB/PTX@RTK) co-loaded an aggregation induced emission (AIE) photosensitizer (TB) and paclitaxel (PTX) was prepared for on-command drug release and synergistic chemo-photodynamic therapy (chemo-PDT). Nano-micelles were characterized for drug loading content, hydrodynamic size, absorption and emission spectra, reactive oxygen species production, and PTX release from micelles. The targeted fluorescence imaging of TB/PTX@RTK micelles and the synergistic anti-tumor efficacy of TB/PTX@RTK micelles-mediated chemo-PDT combined with anti-PD-L1 were assessed both in vitro and in vivo.

**Results:**

The TB/PTX@RTK micelles could specifically accumulate at the tumor site through cRGD-mediated active target and facilitate image-guided PDT for tumor ablation. Once irradiated by light, the AIE photosensitizer of TB could produce ROS for PDT, and the thioketal linker could be cleaved by ROS to precise release of PTX in tumor cells. Chemo-PDT could not only synergistically inhibit tumor growth, but also induce immunogenic cell death and elicit anti-tumor immune response. Meanwhile, chemo-PDT significantly upregulated the expression of PD-L1 on tumor cell surface which could efficiently synergize with anti-PD-L1 monoclonal antibodies to induce an abscopal effect, and establish long-term immunological memory to inhibit tumor relapse and metastasis.

**Conclusion:**

Our results suggest that the combination of TB/PTX@RTK micelle-mediated chemo-PDT with anti-PD-L1 monoclonal antibodies can synergistically enhance systemic anti-tumor effects, and provide a novel insight into the development of new nanomedicine with precise controlled release and multimodal therapy to enhance the therapeutic efficacy of HCC.

**Graphical Abstract:**

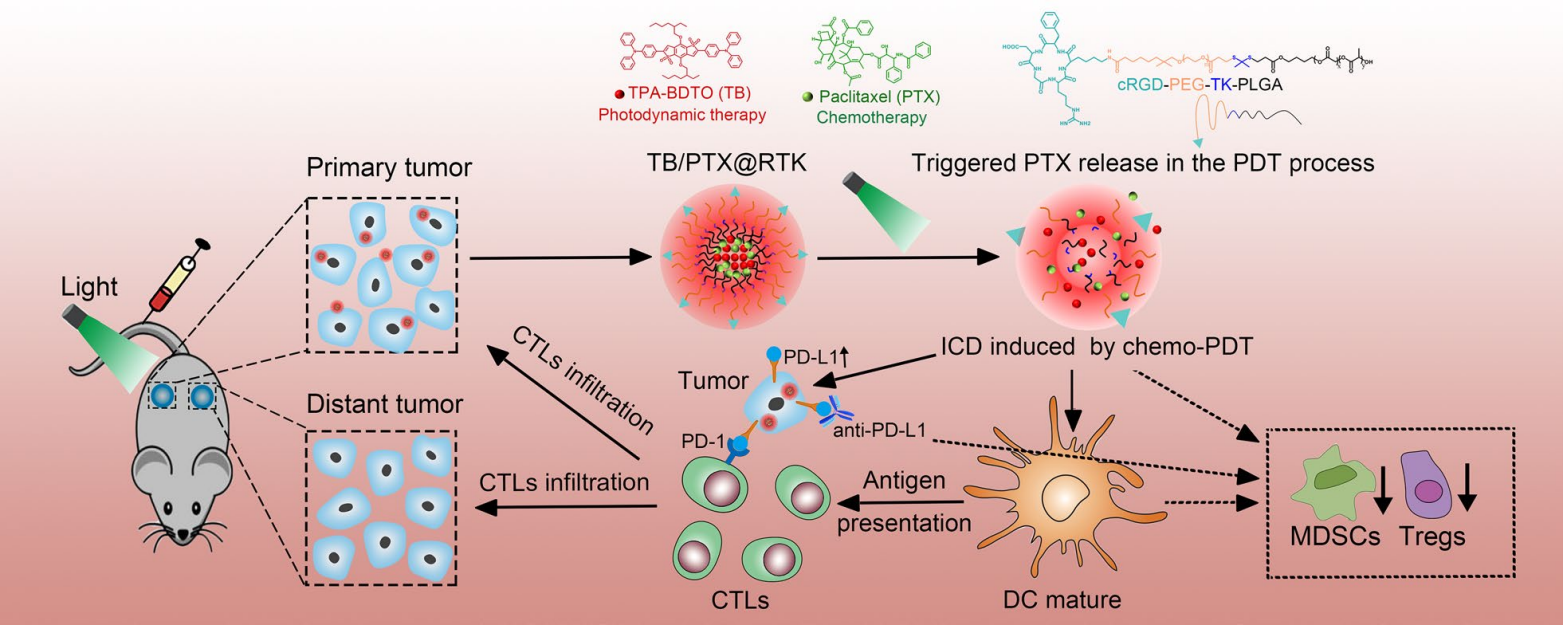

**Supplementary Information:**

The online version contains supplementary material available at 10.1186/s12951-021-01101-1.

## Introduction

Hepatocellular carcinoma (HCC) is the third leading cause of cancer death in the world [1]. Vast majority of patients are diagnosed at advanced stage that lose the chance of radical surgery, and the high probability of recurrence and metastasis leading to more than 90% of cancer-caused deaths is the major challenge in clinical treatment of HCC [2–4]. The current treatment strategies still cannot achieve satisfactory therapeutic effect, especially in unresectable HCC patients. Therefore, how to effectively eradicate the primary tumor and suppress recurrence and metastasis is the key to improve the efficacy of HCC. Recently, immune checkpoint blockade (ICB) therapy has emerged as a promising clinical treatment of advanced and metastatic malignant tumors [5, 6]. ICB therapies, especially programmed cell death protein 1 (PD-1)/programmed cell death ligand 1 (PD-L1) monoclonal antibodies (anti-PD-1/PD-L1), could relieve the immune restriction and restore T lymphocyte function in the tumor microenvironment (TME) [7, 8]. Multiple types of anti-PD-1/PD-L1 monoclonal antibodies were recently approved by the FDA for the treatment of several types of malignancy, including first-line therapy for advanced HCC [9, 10]. However, the objective remission rate of ICB therapy in these patients was less than 20% [7, 11, 12], putatively because of the lack of sufficient release of tumor-associated antigen (TAA) and infiltration of cytotoxic T lymphocytes (CTLs) [13]. In addition, the lack of PD-L1 expression on tumor cells is one of the important reasons for the low response rate to ICB therapy [14, 15]. Hence, more efficient therapy strategy is urgently needed to synergistically improve the efficacy of ICB therapy.

Many other therapeutic methods, such as photodynamic therapy (PDT) and chemotherapy, have been exploited to elicit an anti-tumor immune response [2, 16–22]. PDT employs a combination of photosensitizer, light and oxygen to produce large amounts of reactive oxygen species (ROS) for inducing the immunogenic cell death (ICD) and releasing TAA [18–29]. However, limited by the tissue penetration and tumor hypoxia microenvironment, PDT only shows marginal to moderate therapeutic performance [2, 13, 30]. Besides, although PDT can kill some tumor cells and generate certain levels of anti-tumor immune responses, PDT alone is usually insufficient to inhibit the growth of residual tumor cells in the body after PDT [2, 31, 32]. For chemotherapy, increasing evidences reveal that chemotherapy may increase tumor sensitivity toward immunotherapy [16, 23]. Some chemotherapeutic drugs (e.g., doxorubicin, oxaliplatin, paclitaxel, and so forth) induce ICD through diverse pathways [2, 16, 33, 34], including the concomitant release of neoplastic antigen, the translocation of tumor antigens to the dendritic cell surface, and the secretion of damage-associated molecular patterns (DAMPs) [35–38]. However, poor tumor targeting, multidrug resistance and severe side effects of chemotherapeutic agents have hurdled its application in triggering an immunogenic response in vivo [39, 40]. Combinational therapy that integrates different therapeutic modalities provides an opportunity to achieve better therapeutic efficacy and decreased side effects [16, 40–44]. Based on this concept, combination of PDT and chemotherapy may be a rational strategy for a more powerful activation of immune response and enhanced therapeutic effect with ICB therapy. Besides, PDT or chemotherapy related adaptive immune resistance caused by the up-expression of PD-L1 in tumor cells can be abolished by incorporating anti-PD-L1 monoclonal antibodies into the treatment strategy [13, 23, 24, 45, 46]. Notably, photosensitizer and chemotherapy drugs may have very different physicochemical properties and usually act on different targets, which make a great challenging for efficiently loading and delivering them to their specific targeting sites. Therefore, it is necessary to design an ideal drug delivery system that enable stable transportation of anti-tumor drugs in the circulation without leakage before reaching the tumor site, and precise release drugs in subcellular localization.

Self-assembly polymeric nanocarriers-based drug delivery systems (nano-DDSs) have received considerable attention for designing targeted and smart stimulus-responsive anti-tumor drug treatment strategies [40, 47–51]. Nano-DDSs with high drug loading content can overcome the disadvantage of high hydrophobic drugs, elongate blood circulation time, and improve the bioavailability of drugs. By surface modification of specific ligands, nano-micelles can achieve specific tumor targeting ability [9, 13, 31, 32]. Moreover, the on-command drug release from stimuli-triggered disassembly of nanocarriers in designated time and space could be achieved by some external stimulus, such as light [16, 25, 40]. For example, ROS-sensitive nanocarriers loaded with photosensitizers can not only play the role of PDT but also cleave ROS-sensitive groups to release drugs under light irradiation [16, 40]. Despite being promising, the design of versatile nano-DDSs with on-command drug delivery and drug release for highly efficient anti-tumor therapy remains a formidable challenge.

**Scheme 1.**
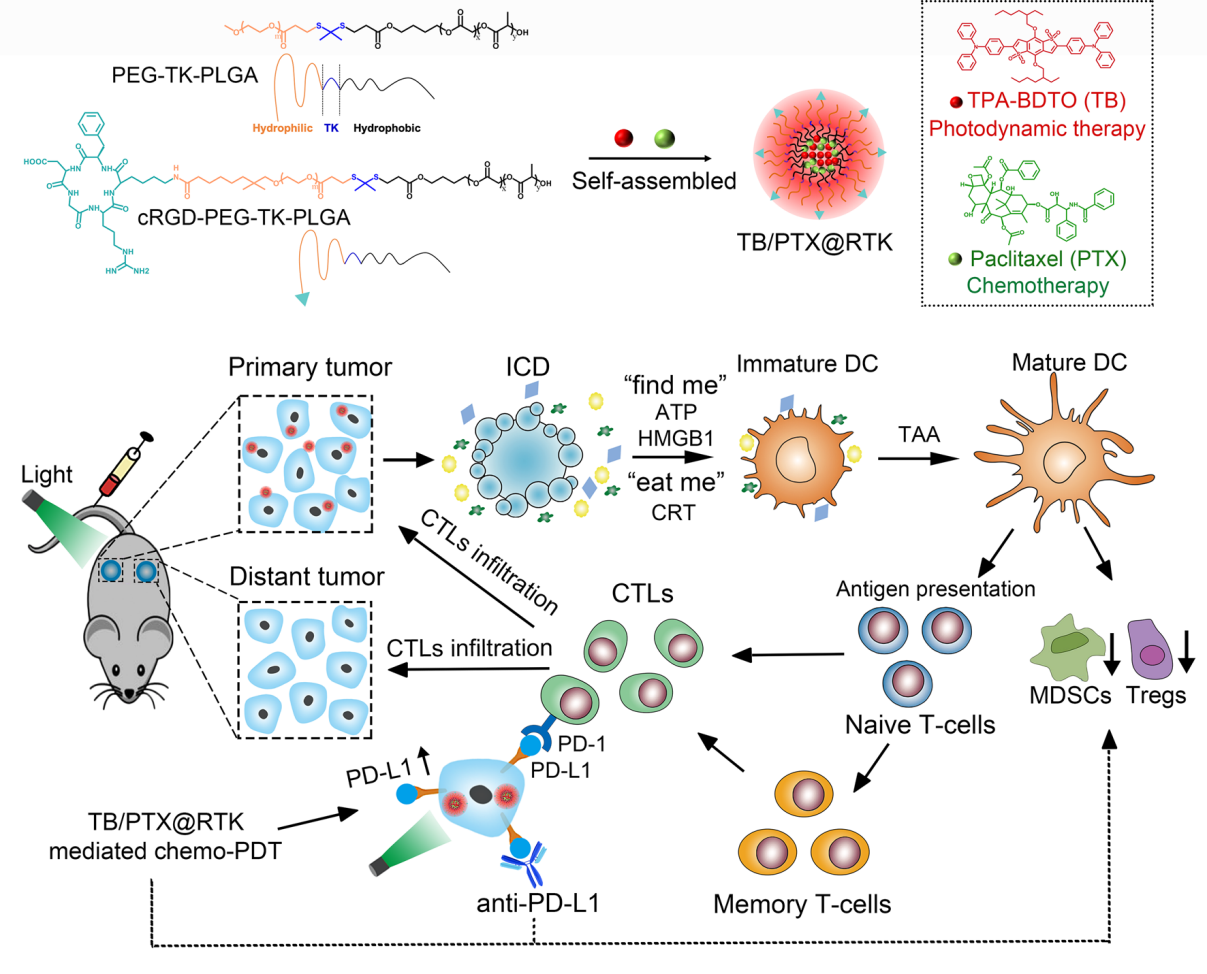
Schematic illustration of TB/PTX@RTK micelles mediated chemo-PDT to potentiate immune checkpoint blockade therapy. TB/PTX@RTK micelles enter tumor cells through active targeting. Under light irradiation, photosensitizer TB in micelles uses oxygen to generate ROS and PTX is immediately released from the micelles, causing ICD and releasing TAA for dendritic cells (DCs) maturation, migration, and cytotoxic T cell activation. Meanwhile, TB/PTX@RTK mediated chemo-PDT could significantly upregulate the expression of PD-L1 on tumor cells and reverse the immunosuppressive tumor microenvironment. Combination chemo-PDT with anti-PD-L1 antibody induces complete tumor regression of primary tumors and efficiently inhibits distant metastatic tumors by triggering anti-tumor immune responses. Finally, immunological memory established by chemo-PDT with PD-L1 blockade efficiently prevents tumor recurrence by rechallenged tumor cells

Here, we designed a light-triggered disassembly of nanoplatform (TB/PTX@RTK) co-loaded an aggregation induced emission (AIE) photosensitizer (TB) and paclitaxel (PTX) for on-command drug release and synergistic chemo-PDT [23, 31, 32]. The nanocarrier originates from a ROS-sensitive thioketal (TK) linkage-bridged diblock copolymer of PEG with polylactic acid-glycolic acid (PLGA) (PEG-TK-PLGA) [52]. The cRGD peptide is coupled with NHS-PEG-TK-PLGA through an amidation reaction, and then through the self-assembly of cRGD-PEG-TK-PLGA and PEG-TK-PLGA, excellent tumor targeting can be obtained on the prepared micelles of cRGD-modified PEG-TK-PLGA (RTK) [31, 32]. As an AIE photosensitizer, TB has stronger photosensitive properties and overcomes the aggregation-caused quenching effect [53, 54]. Under light irradiation, TB/PTX@RTK micelles have a more powerful tumor killing and anti-tumor immunity activation effects. Meanwhile, chemo-PDT could significantly upregulate the expression of PD-L1 on tumor cells which could efficiently synergize with anti-PD-L1 antibodies to induce an abscopal effect, and establish long-term immunological memory to inhibit the recurrence and metastasis of tumor (Scheme 1). This study might provide novel insight into the development of new nanomedicine with precise controlled release and multimodal therapy to enhance the therapeutic efficacy of HCC.

## Results and discussion

### Synthesis and characterization of the ROS-sensitive micelles with tumor targeting function

**Fig. 1:**
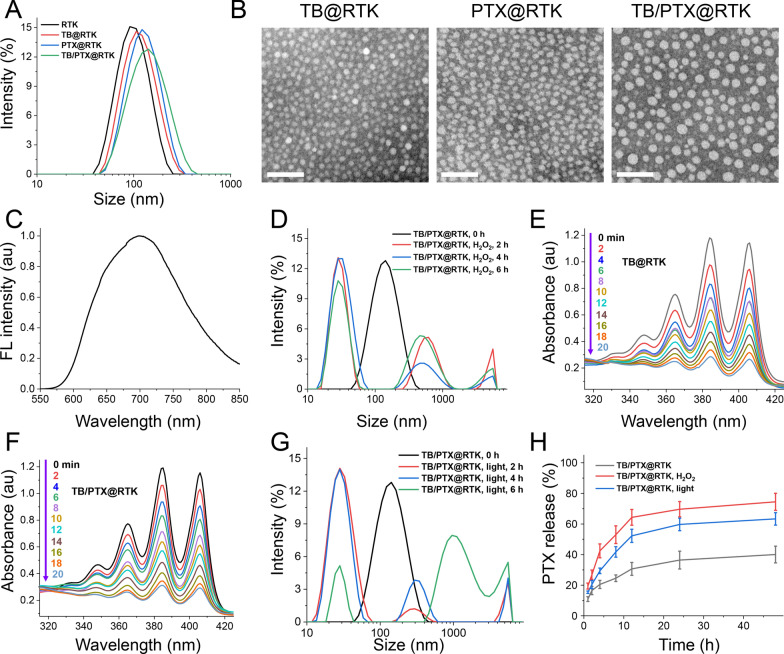
Preparation and characterization of RTK, TB@RTK, PTX@RTK and TB/PTX@RTK micelles. **A** Hydrodynamic size distribution of RTK, TB@RTK, PTX@RTK and TB/PTX@RTK micelles. **B** The TEM images of TB@RTK, PTX@RTK and TB/PTX@RTK micelles. Scale bar: 100 nm. **C** FL spectra of TB/PTX@RTK micelles in PBS (Ex = 530 nm). **D** Hydrodynamic size distribution of TB/PTX@RTK micelles were treated with 10 mM H_2_O_2_ for 0 h, 2 h, 4 h and 6 h, respectively. UV–vis absorption spectrum of TB@RTK (**E**) and TB/PTX@RTK (**F**) micelles upon light irradiation (white light, 100 mW/cm^2^), respectively. **G** Hydrodynamic size distribution of TB/PTX@RTK micelles upon light irradiation (white light, 100 mW/cm^2^, 10 min) after 2 h, 4 h and 6 h, respectively. **H** In vitro release of PTX from TB/PTX@RTK micelles in PBS (pH 7.4, 0.1 M) containing 0.1% (w/v) Tween 80 at 37 ℃ or H_2_O_2_ (10 mM) or light irradiation

The AIE photosensitizer of TPABDTO (TB) was synthesized referred to the previous report [52, 53]. The cRGD-PEG-TK-PLGA was obtained by amidation reaction between -NHS and -NH_2_ from NHS-PEG-TK-PLGA and cRGD (RK-5) (Additional file 1: Figure S1) [54]. The ROS-sensitive micelles with tumor targeting function of RTK was prepared by dialysis method using cRGD-PEG-TK-PLGA and PEG-TK-PLGA. The hydrophobic AIE photosensitizer of TB and the chemotherapeutic drug of PTX loaded micelles with targeting function were prepared by the same method, the PEG-TK-PLGA and cRGD-PEG-TK-PLGA were used as carriers material, that is TB@RTK, PTX@RTK and TB/PTX@RTK. The hydrodynamic sizes of RTK, TB@RTK, PTX@RTK and TB/PTX@RTK were 91.39 ± 0.2 nm (PDI = 0.182 ± 0.017), 100.4 ± 2.0 nm (PDI = 0.213 ± 0.009), 112.9 ± 4.5 nm ((PDI = 0.223 ± 0.019) and 121.2 ± 1.2 nm (PDI = 0.219 ± 0.015), respectively (Additional file 1: Table S1 and Fig. 1A). The uniform spherical shape of TB@RTK, PTX@RTK and TB/PTX@RTK micelles was observed by transmission electron microscopy (TEM) (Fig. 1B). It may be that the sample is in a dry state when testing the TEM, which causes the shrinkage of PEG, and the corresponding hydrodynamic size are therefore larger than their size of TEM. In addition, the drug loading content (DLC) of TB reached up to 7.43% and 7.83% for TB@RTK and TB/PTX@RTK micelles, the DLC of PTX were 6.15% and 5.37% for PTX@RTK and TB/PTX@RTK micelles, respectively. As shown in Fig. 1C, TB/PTX@RTK micelles showed relatively strong emission peaking at approximately 684 nm in aqueous solution, which could be used for self-tracking. Due to the structure of PEG-TK-PLGA containing the ROS-triggered TK group, the tumor-targeting micelles of TB/PTX@RTK could show the expected ROS cleavage characteristic to realize the controlled release of the drug in tumor cells. In order to verify that the ROS-sensitive of TB/PTX@RTK, its size change in H_2_O_2_ (simulated oxidation environment) was determined by dynamic light scattering (DLS). After incubation in H_2_O_2_ for different time, the size of TB/PTX@RTK micelles was changed from single peak to multiple peak for 2 h, and the PDI of TB/PTX@RTK micelles was changed from 0.219 to 0.489 (Fig. 1D). Then, the ROS generation ability of AIE photosensitizers of TB loaded micelles of TB@RTK and TB/PTX@RTK under light irradiation was evaluated using 9,10-Anthracenediyl-bis(methylene)dimalonic acid (ABDA) as indicator. As shown in Fig. 1E and F, the absorbance intensity of TB@RTK and TB/PTX@RTK micelles was rapidly decreased during irradiation. These data clearly showed that the self-assembled amphiphilic micelles could efficiently generate ROS under light irradiation for AIE photosensitizers loaded micelles. Furthermore, the hydrodynamic size distribution of TB/PTX@RTK micelles is more chaotic by DLS, which may be due to the stimulation of the TK structure of the carrier by ROS generated by light irradiation (Fig. 1G). This meant that the light administered during PDT treatment not only excited the AIE photosensitizer of TB to produce ROS, but also activates the release of PTX spatially. More importantly, drug controlled release experiments also demonstrated that the TB/PTX@RTK micelles released the loaded PTX in a ROS-dependent manner. PTX released from TB/PTX@RTK micelles was accelerated at the condition of H_2_O_2_ (10 mM), and about 75% of PTX released from TB/PTX@RTK micelles for 48 h (Fig. 1H). As expected, it was found that light-triggered PTX release occurred after light irradiation (white light, 100 mW/cm^2^, 10 min), and about 64% of PTX was released from TB/PTX@RTK micelles under light irradiation for 48 h. In contrast, TB/PTX@RTK micelles exhibited relatively slow PTX release under the condition without any treatment. These results indicated that the development of ROS-sensitive TB/PTX@RTK micelles with AIE photosensitizers was an effective promising strategy to promote the release of PTX under light irradiation.

### Targeted imaging and subcellular localization of tumor cells by TB/PTX@RTK micelles in vitro

**Fig. 2:**
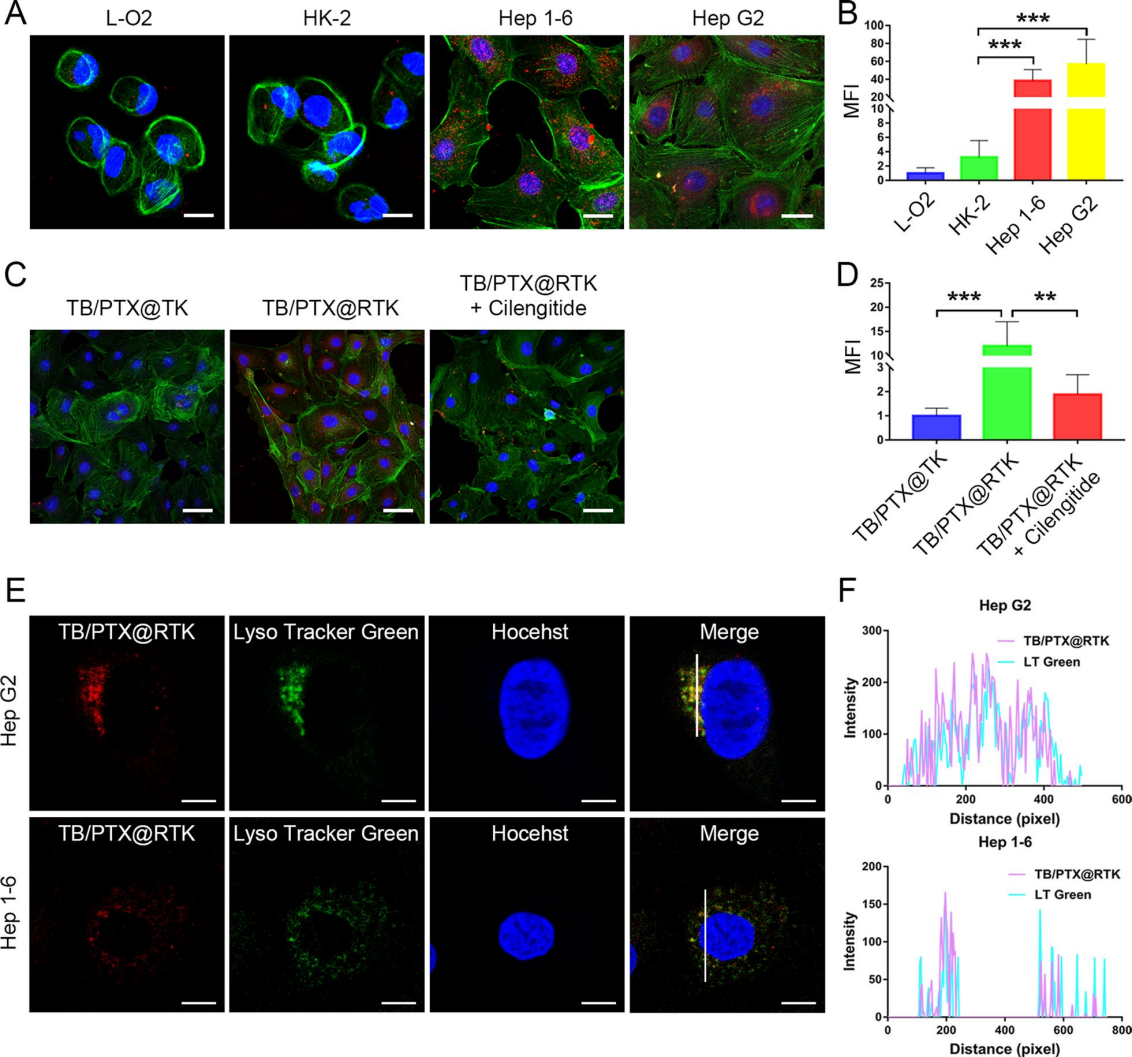
Targeted imaging and subcellular localization of tumor cells by TB/PTX@RTK micelles in vitro. **A**, **B** CLSM images and relative semi-quantitative analysis of the fluorescence intensity of L-O2, HK-2, Hep G2 and Hep 1–6 cells after incubated with the micelles (5 μg/mL) for 4 h. Red fluorescence is emitted by the micelles. Green fluorescence and blue fluorescence label the cytoskeleton and the nuclei, respectively. Scale bar: 20 μm. **C**, **D** Confocal images and semi-quantitative analysis of Hep G2 after incubation with TB/PTX@TK or TB/PTX@RTK micelles (5 μg/mL) for 4 h without or with pretreatment with cilengitide. Scale bars: 50 μm. **E**, **F** Confocal images and co-localization analysis of TB/PTX@RTK micelles with lysosomes. Scale bar: 10 μm

Our previous research has confirmed that integrin α_ν_β_3_, as cRGD receptor, was highly expressed in HCC cells [31]. To demonstrate the active targeted ability of cRGD modified micelles to HCC cells, Hep 1–6 and Hep G2 cells were treated with TB/PTX@RTK micelles. Normal tissue cell lines L-O2 and HK-2 with low integrin α_ν_β_3_ expression were used as controls. After being co-incubated with the micelles (5 μg/mL) for 4 h, cells were imaged using a confocal laser scanning microscopy (CLSM). As shown in Fig. 2A, B, the fluorescence intensity emitted by the AIE photosensitizer of TB in Hep G2 and Hep 1–6 cells was stronger than that in L-O2 and HK-2 cells, indicating that tumor cells exhibited more uptake of TB/PTX@RTK micelles. Besides, when compared with TB/PTX@TK micelles that without cRGD modification, Hep G2 cells exhibited more uptake of TB/PTX@RTK (Fig. 2C, D). To further evaluate the specificity of TB/PTX@RTK micelles towards HCC cells, competitive binding test was performed. As anticipated, Hep G2 cells pretreated with cilengitide, an integrin α_ν_β_3_ inhibitor, prior to micelles incubation exhibited a dramatically reduced uptake of TB/PTX@RTK micelles (Fig. 2C, D). In addition, flow cytometry analysis was used to assess the specificity of TB/PTX@RTK micelles targeting HCC cells, and the results were in accordance with the relative semi-quantitative fluorescence analysis based on CLSM imaging (Additional file 1: Figure S2). To explore the distribution of TB/PTX@RTK in tumor cells, LysoTracker Green was used to label the lysosomes. As shown in Fig. 2E, F, the subcellular localization of TB/PTX@RTK micelles almost coincided with that of lysosomes, which suggested that micelles mainly entered cells through endocytosis. Based on the cell uptake experiment in vitro, the fluorescence intensity in cells increased with the extension of incubation time, and the co-localization showed that the fluorescence mainly accumulates in lysosomes indicating that the subcellular localization of micelles after endocytosis was mainly located in lysosomes (Additional file 1: Figure S3). These above results indicated that TB/PTX@RTK micelles had the ability to target HCC cells, and mainly through ligand-receptor mediated active endocytosis.

### Fluorescence imaging, biodistribution analysis and pharmacokinetics of TB/PTX@RTK micelles in vivo

Prior to applications in vivo, hemolysis tests in vitro were performed to assess the biocompatibility of TB/PTX@RTK micelles. As shown in Additional file 1: Figure S4A, no detectable hemolysis occurred in TB/PTX@RTK micelles solutions under the studied time and concentration range. Furthermore, no obvious morphological change of RBCs was observed in TB/PTX@RTK micelles as shown in Additional file 1: Figure S4B. These results suggested that TB/PTX@RTK micelles were hemocompatible and could be safely administered intravenously.

**Fig. 3:**
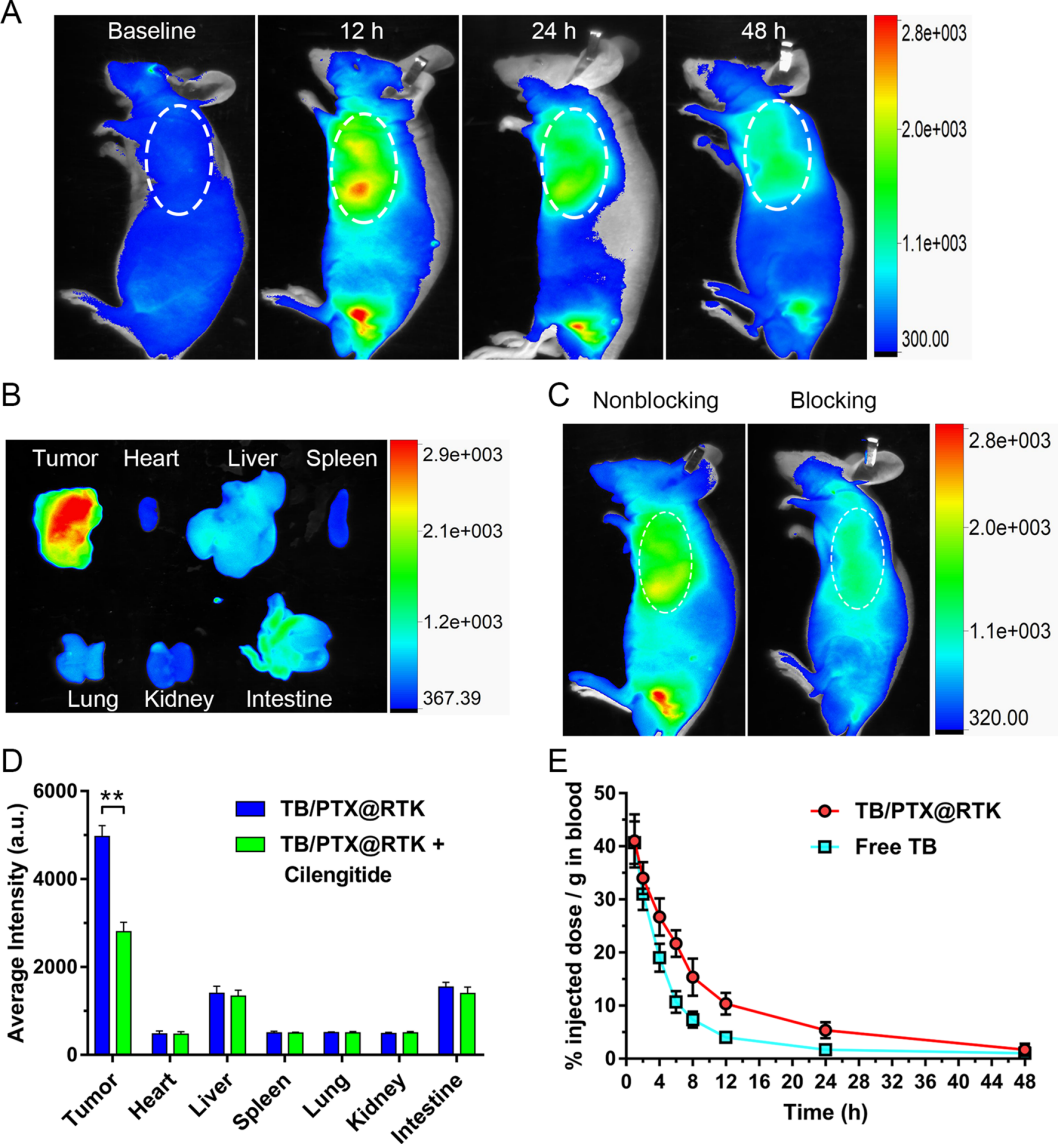
Fluorescence imaging, biodistribution analysis and pharmacokinetics of TB/PTX@RTK micelles in vivo. **A** Fluorescent images of the Hep 1–6 tumor-bearing Balb/c nude mice at different time points after injection of TB/PTX@RTK micelles (5 mg/kg). The white dotted ellipse indicates tumor sites. **B** Ex vivo fluorescence imaging of tumor tissues and major organs from tumor-bearing mice 12 h after intravenous injection of micelles. **C** Biodistribution of TB/PTX@RTK micelles in tumor-bearing mice 12 h after injection of TB/PTX@RTK micelles (5 mg/kg) without or with pretreatment with cilengitide. The white dotted ellipse indicates tumor sites. **D** Quantitative analysis of organ distribution of TB/PTX@RTK micelles in tumor-bearing mice with or without pretreatment with cilengitide. Data represent mean ± SD (n = 3). **p < 0.01. **E** Pharmacokinetics of free TB and TB/PTX@RTK micelles in the blood. Data represent mean ± SD (n = 3)

In order to demonstrate that TB/PTX@RTK micelles could achieve targeted tumor accumulation in vivo, Hep 1–6 tumor-bearing Balb/c nude mice models were established to examine tumor accumulation and semi-quantity the biodistribution of TB/PTX@RTK micelles. Immediately following systemic administration, TB/PTX@RTK micelles were distributed widely throughout the body and then accumulated in the major organs and tumor tissue (Fig. 3A, B). Selective high-level accumulation of TB/PTX@RTK micelles in the tumor tissue at 12 h was confirmed by ex vivo fluorescence measurements (Fig. 3A, B). Next, in order to further evaluate the specificity of TB/PTX@RTK micelles towards HCC cells, competitive binding test was performed in vivo. As anticipated, Hep 1–6 tumor-bearing Balb/c nude mice pretreated with cilengitide prior to micelles injection exhibited a dramatically reduced uptake of TB/PTX@RTK micelles (Fig. 3C). Meanwhile, to evaluate the biodistribution of TB/PTX@RTK micelles in vivo, the mice were sacrificed 12 h after being injected with the micelles and tumor tissues and major organs were harvested. The fluorescence intensity of the major organs and tumor tissues was measured by an In-Vivo FX PRO for semi-quantitative biodistribution analysis. As shown in Fig. 3D and Additional file 1: Figure S5, the administration of cilengitide did not significantly influence the biodistribution of TB/PTX@RTK micelles in the major organs. However, the fluorescence intensity of the tumor tissues in the non-blocking group was obviously higher than that in the blocking group. Together, these results demonstrated that cRGD modification significantly enhanced the targeting ability of TB/PTX@RTK micelles in vivo.

It has been showed that the longer the nanomedicines circulate in the blood, the more likely they are to accumulate at the tumor site [55]. To verify the prolonged retention of TB/PTX@RTK micelles in bloodstream, pharmacokinetic studies were performed. As shown in Fig. 3E, the clearance half-life (t_1/2_) of TB/PTX@RTK micelles (~ 12.5 h) in bloodstream was significantly longer than that of free TB (~ 4.0 h), possibly due to the reduced uptake of PEG-modified micelles by the reticuloendothelial system, which provided sufficient time for TB/PTX@RTK micelles to accumulate at the tumor site [31, 54].

### ROS generation, light-triggered PTX release and synergistic chemo-PDT in vitro

**Fig. 4:**
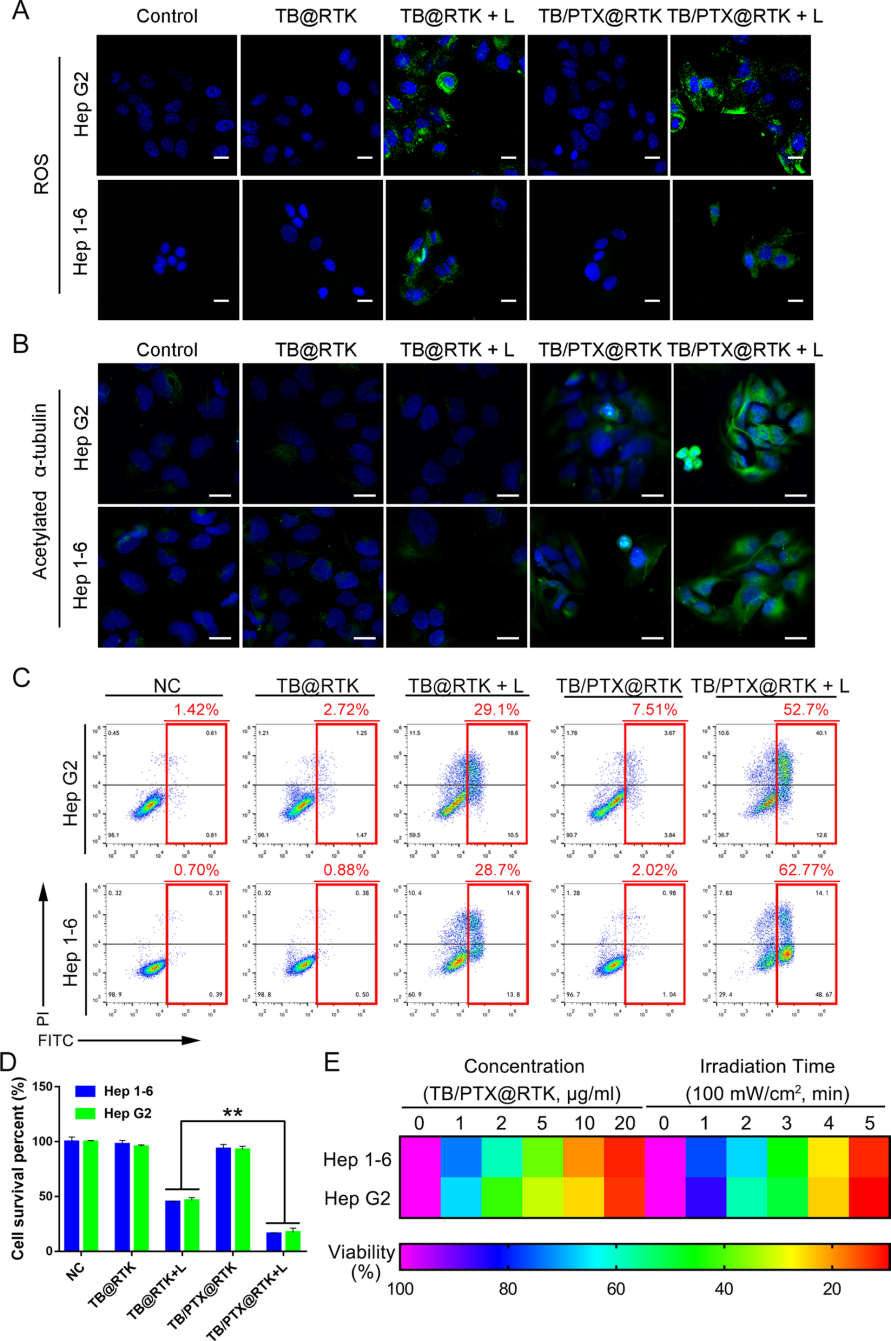
ROS generation, intracellular PTX release and synergistic chemo-PDT in vitro. **A** Detection of the intracellular ROS generation induced by TB-mediated PDT in Hep 1–6 and Hep G2 cells. Scale bar: 20 μm. **B** Immunofluorescent staining of acetylated α-tubulin in Hep 1–6 and Hep G2 cells after treatment with PBS (negative control), TB@RTK, TB@RTK + light, TB/PTX@RTK, TB/PTX@RTK + light. Scale bar: 20 μm. **C** Flow cytometric analysis of cell apoptosis and necrosis in Hep 1–6 and Hep G2 cells after treatment with PBS (negative control), TB@RTK, TB@RTK + light, TB/PTX@RTK, TB/PTX@RTK + light. **D** Cell viability following treatment with PBS (negative control), TB@RTK, TB@RTK + light, TB/PTX@RTK, TB/PTX@RTK + light. **E** Cell viability after treatment with different concentration of TB/PTX@RTK micelles or time of light irradiation

According to our design, light triggered TB/PTX@RTK micelles could produce high concentration of ROS in localized area, that on hand for PDT and the other hand for inducing the disassembly of micelles and drug release. Based on this assumption, we thus firstly detected the ROS production of TB/PTX@RTK micelles in HCC cells under light irradiation. We found that large amounts of ROS were generated in HCC cells treated with TB@RTK and TB/PTX@RTK micelles under light irradiation, indicating that light could trigger efficient ROS generation by TB (Fig. 4A and Additional file 1: Figure S6). Release of PTX is the premise of TB/PTX@RTK micelles to act chemotherapeutic effect. We thus investigated the PTX release from TB/PTX@RTK micelles in Hep G2 cells under light irradiation. Previous studies have confirmed that PTX stabilized microtubules in their polymerized form leading to malignant tumor cell death [56]. Then, we assessed two markers of microtubule stability, acetylated α-tubulin and detyrosinated α-tubulin to determine whether generating ROS would promote PTX release from micelles [56]. Interestingly, in our research, the levels of acetylated α-tubulin and detyrosinated α-tubulin dramatically increased in TB/PTX@RTK with light group, indicating that PTX was successfully released from the micelles (Fig. 4B, Additional file 1: Fig. S7–S9). These results suggested that large amounts of ROS produced by PDT were conducive to the release of PTX from TB/PTX@RTK micelles.

We further explored the synergistic therapeutic effect of PTX-mediated chemotherapy and photosensitizer TB-mediated PDT. In our study, we found that the light used for PDT (540 nm, 100 mW/cm2, 3 min) alone had no effect on ROS production and cell activity (Additional file 1: Figure S10). The focus of our study was to evaluate whether chemo-PDT had a stronger ability to kill tumor cells and activate anti-tumor immunity when compared to single therapy model. To simplify the groups set, "light only" group was not included in the next study, and this did not affect the conclusions of the study [40, 44]. As shown in Fig. 4C, D and Additional file 1: Figure S11, neither TB@RTK nor TB/PTX@RTK micelles alone displayed obvious cytotoxic effects. However, in combination with light irradiation, TB/PTX@RTK micelles displayed significantly stronger cell killing effect than TB@RTK micelles. The difference suggested that the ROS generated by TB/PTX@RTK micelles under light was sufficient to trigger rapid release of chemotherapeutics PTX, which effectively synergized with TB-mediated PDT to kill HCC cells (Fig. 4C, D). Viable and dead cells fluorescent staining using Calcein-AM and PI were performed to assess chemo-PDT-induced cell death. Cell death occurred only in areas where both TB/PTX@RTK micelles and light irradiation were present, and few cell death was detected in non-irradiated areas (Additional file 1: Figure S12). These results further indicated that the cytotoxicity of TB/PTX@RTK micelles was mainly controlled by light irradiation. As shown in Fig. 4E, with increasing concentration of TB/PTX@RTK micelles or time of light irradiation, cell viability declined more rapidly, indicating that the therapeutic efficiency of TB/PTX@RTK-mediated chemo-PDT was concentration- and light irradiation time-dependent. In addition, flow cytometry analysis using the Annexin V-FITC/PI Apoptosis Assay Kit showed that apoptotic cells increased rapidly with increasing concentration of TB/PTX@RTK micelles or time of light irradiation (Additional file 1: Figure S13). These results indicated that the therapeutic efficiency of TB/PTX@RTK-mediated chemo-PDT changed with variations in laser irradiation exposure time and micelle concentration.

### Effects of TB/PTX@RTK-mediated chemo-PDT in vivo

**Fig. 5:**
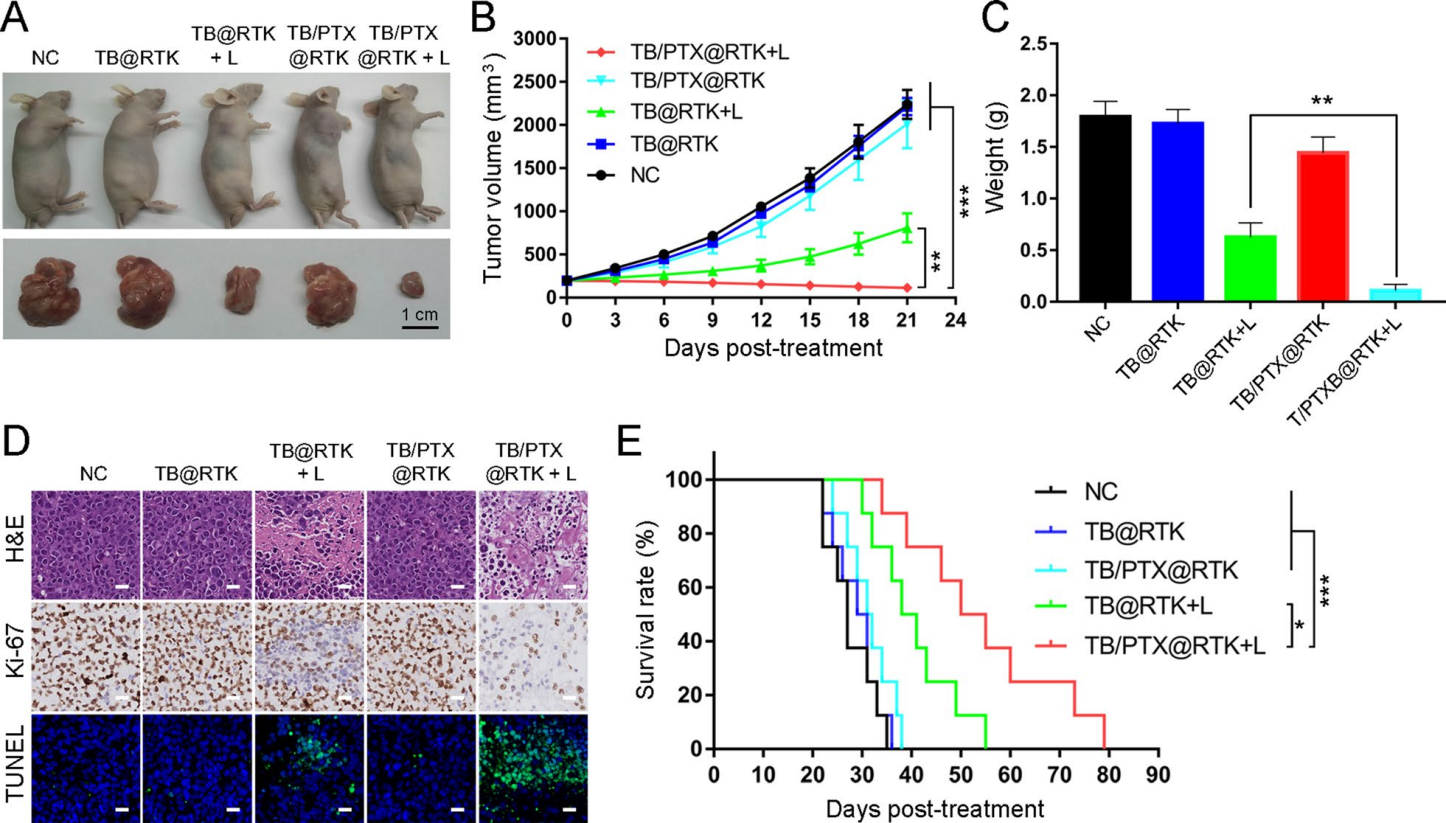
Anti-tumor efficacy of TB/PTX@RTK micelles in vivo. **A** Representative images of tumor-bearing mice and harvested tumors after various treatments. Scale bar: 1 cm. **B** Tumor volume at indicated time points after different treatments (n = 5 per group). Data represent mean ± SD. **C** Tumor weight at 21 days after different treatments. Data represent mean ± SD. **D** H&E staining for histological changes in Hep 1–6 tumor tissues after different treatments (upper row). Ki-67 expression detected by IHC in tumor tissues sections after different treatments (middle row). TUNEL assay for apoptosis levels in Hep 1–6 tumor tissues after different treatments (lower row). Scale bar: 20 μm. **E** Survival curves of Hep 1–6 tumor-bearing C57BL/6 mice in different treatment groups (n = 8 per group). *P < 0.05, ***P < 0.001

C57BL/6 mice with subcutaneous Hep 1–6 tumors were used as the animal model to evaluate the therapeutic effect of TB/PTX@RTK-mediated chemo-PDT. Tumor-bearing C57BL/6 mice were divided into five groups (n = 18 per group) and treated with PBS (negative control), TB@RTK, TB@RTK + light, TB/PTX@RTK, or TB/PTX@RTK + light, respectively. The treatment effects of different treatments were dynamically evaluated by measuring tumor volume and survival. In the PBS, TB@RTK, and TB/PTX@RTK groups, tumor growth was not obviously inhibited (Fig. 5A, B). As shown in Fig. 5B, tumor growth in the TB@RTK + light group was inhibited in the early stages, but later the tumor appeared to recurrence, manifesting that PDT alone was not enough to completely inhibit tumor progression. Notably, the tumor suppressive effect of TB/PTX@RTK-mediated chemo-PDT was significantly better than that of PDT alone (Fig. 5B). On day 21 after different treatments, tumor tissues were harvested and weighed (n = 5 per group), and the representative tumor-bearing mice and their tumor tissues were photographed. As shown in Fig. 5C, a significant difference in tumor weight was observed between the TB/PTX@RTK + light group and the other four groups. To further assess the therapeutic efficacy of TB/PTX@RTK-mediated chemo-PDT, hematoxylin–eosin (H&E) staining, TUNEL assay and Ki-67 immunohistochemistry (IHC) were performed on tumor tissues at 3 day after different treatments (n = 5 per group). It was found that tumor cells in the PBS (negative control), TB@RTK and TB/PTX@RTK groups were dense (Fig. 5D). In contrast, tumor cells in TB/PTX@RTK + light group showed the most nuclei absence, manifesting that a great deal of tumor cell was destroyed during the TB/PTX@RTK-mediated chemo-PDT process. In addition, the survival time of the tumor-bearing mice in the chemo-PDT group (n = 8 per group) was significantly prolonged (Fig. 5E). Together, these results suggested that TB/PTX@RTK-mediated chemo-PDT might serve as an effective strategy for anti-tumor therapy.

### ICD induction, immune activation and adaptive immune resistance of TB/PTX@RTK-mediated chemo-PDT

**Fig. 6:**
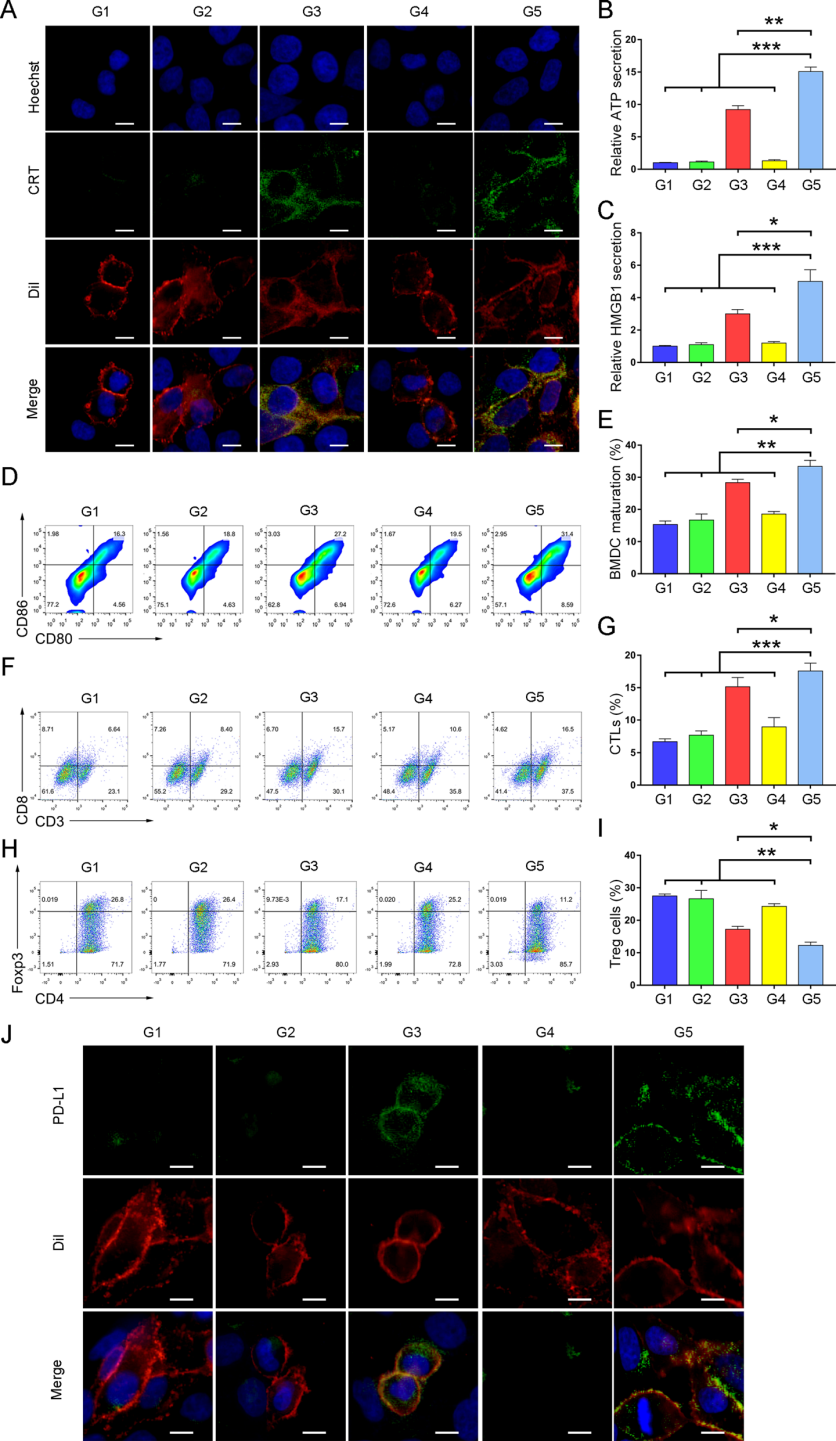
ICD induction, immune activation and adaptive immune resistance of TB/PTX@RTK-mediated chemo-PDT (G1: PBS; G2: TB@RTK; G3: TB@RTK + L; G4: TB/PTX@RTK; and G5: TB/PTX@RTK + L). **A** Immunofluorescence imaging of CRT expression on the membrane surface of Hep 1–6 cells after different treatments. DiI indicates the cytomembrane. Scale bars: 10 μm. Extracellular ATP (**B**) and HMGB1 (**C**) levels after different treatments. *P < 0.05, **P < 0.01, ***P < 0.001. (**D**–**I**) Representative plots of flow cytometric analysis and statistical analysis of mature BMDCs (CD11c^+^CD80^+^CD86^+^; **D** and **E**), CTLs (CD45^+^CD3^+^CD8^+^; **F** and **G**) and Treg cells (CD3^+^CD4^+^Foxp3^+^; H and I) in the tumors. **J** Immunofluorescence imaging of membrane PD-L1 proteins on Hep 1–6 cells after different treatments. DiI indicates the cytomembrane. Scale bars: 10 μm

Encouraged by the promising results of anti-tumor efficacy, we next explored the effect of TB/PTX@RTK mediated chemo-PDT on anti-tumor immunity. It was clarified that the immune profile of ICD pathways was defined by a cluster of molecules called DAMPs, such as calreticulin (CRT), heat shock protein 70 (HSP70), adenosine triphosphate (ATP), and high mobility group box 1 (HMGB1), and so on [20, 35, 36, 38]. Chemo-PDT stimulated the constellation of alterations in the dying tumor cells, such as HMGB1 efflux, HSP70 exposure and ATP secretion as “find me” signal, CRT exposure as an “eat me” signal to attract and activate antigen-presenting cells, leading to activation of adaptive anti-tumor immunity. Hence, we evaluated the ability of TB/PTX@RTK micelles to efficiently induce two ICD-inducing modalities after light irradiation in vitro. We found that TB/PTX@RTK-mediated chemo-PDT did not change the total CRT content of HCC cells, but significantly increased the expression of CRT on the cell membrane surface more than PDT alone (Fig. 6A, Additional file 1: Fig. S14). The reasonable explanation might be that ICD promoted the migration of CRT from the endoplasmic reticulum (ER) to the surface of the cell membrane [20]. As "find me" signal, the extracellular secretion of HMGB and ATP, and the expression of HSP70 on the cell membrane were more efficiently induced by TB/PTX@RTK-mediated chemo-PDT to attract antigen-presenting cells into the tumor (Fig. 6B, C, Additional file 1: Fig. S15–S17). These results substantiated that TB/PTX@RTK-mediated chemo-PDT with two ICD-inducing modalities was more powerful to induce ICD than single therapy model.

DCs are the main antigen-presenting cells that present antigens to CD8^+^ CTLs and further activate CTLs [9, 19]. We then examined the effects of chemo-PDT-induced ICD on the maturation of bone marrow-derived DCs (BMDCs). Interestingly, we observed the significant maturation of BMDCs, as indicated by increased CD11c^+^CD80^+^CD86^+^ cells in TB/PTX@RTK + light group (Fig. 6D, E). To further assess the maturation of DCs after chemo-PDT in vivo, the mice were euthanized 3 day after different treatments and ELISA was used to detect the serum IL-12 secreted by mature DCs upon TAA stimulation [19]. There was a significant upregulation of IL-12 levels in TB/PTX@RTK + light group compared with other groups (Additional file 1: Figure S18), confirming that DC maturation was significantly induced by TB/PTX@RTK-mediated ICD.

Next, we explored whether TB/PTX@RTK-mediated chemo-PDT could effectively remodel the TME. Therefore, we further studied the anti-tumor CTL (CD45^+^CD3^+^CD8^+^) and immunosuppressive Treg cell (CD3^+^CD4^+^Foxp3^+^) populations of tumor after different treatments. Compared with PDT alone, TB/PTX@RTK-mediated chemo-PDT significantly increased the infiltration of CTLs and decreased the infiltration of Treg cells in the tumor (Fig. 8F–I). In addition, we studied CTLs activation by measuring the levels of IFN-γ produced by activated CTLs in tumor tissues [16, 24]. There was a significant upregulation of IFN-γ levels in TB/PTX@RTK + light group compared with the other four groups (Additional file 1: Figure S19), confirming that CTLs were effectively activated by TB/PTX@RTK-mediated chemo-PDT. These results suggested that TB/PTX@RTK-mediated chemo-PDT enhanced anti-tumor immunity.

However, recent studies reported that PDT and multiple of chemotherapeutic agents could elevate the expression of PD-L1 on tumor cells. Membrane PD-L1 is able to bind with PD-1 on the surface of CTLs and inhibit its function. In addition, it was reported that the activated adaptive immune resistance in response to therapy was positively correlated with tumor recurrence and metastasis [13, 24, 57]. In this study, we found that the expression of PD-L1 on the cell membrane in TB/PTX@RTK + light group was increased the most compared with other groups (Fig. 6J, Additional file 1: Fig. S20, S21). In order to further clarify whether TB-mediated PDT or PTX-mediated chemotherapy contributed the most to the upregulation of PD-L1, free PTX group was included in the study. Compared with PTX-mediated chemotherapy, TB-mediated PDT contributed more to the upregulation of PD-L1 (Additional file 1: Figure S22). These results suggested that TB/PTX@RTK-mediated chemo-PDT could synergistically induce the upregulation of PD-L1 and activate adaptive immune resistance. The increased expression of PD-L1 on tumor cells is like a "double-edged sword". Although it inhibits the function of CTLs, the current researches showed that the high expression of PD-L1 was positively correlated with tumor response to ICB therapy. Therefore, we assumed that the combination therapy with anti-PD-1/PD-L1 monoclonal antibodies could effectively reverse adaptive immune tolerance and potentiate the anti-tumor efficacy of ICB.

### The potential mechanism of ICD induced by TB@RTK micelle-mediated PDT

As TB@RTK micelle-mediated PDT has been proved as an effective treatment to incite tumor cell death and elicit the immunogenicity of dying tumor cells, there is growing interest in exploring the potential mechanisms of TB@RTK micelles mediated ICD of tumor cells. Previous studies have reported that high production of ROS produced by photosensitizers located in lysosomes could damage lysosomes and further induce apoptosis through the mitochondria-mediated pathway [58]. Considering that the subcellular localization of TB@RTK micelles almost coincided with that of lysosomes, we thus evaluated whether mitochondrial mediated apoptosis pathways were involved in the ICD induced by TB@RTK micelle-mediated PDT.

**Fig. 7:**
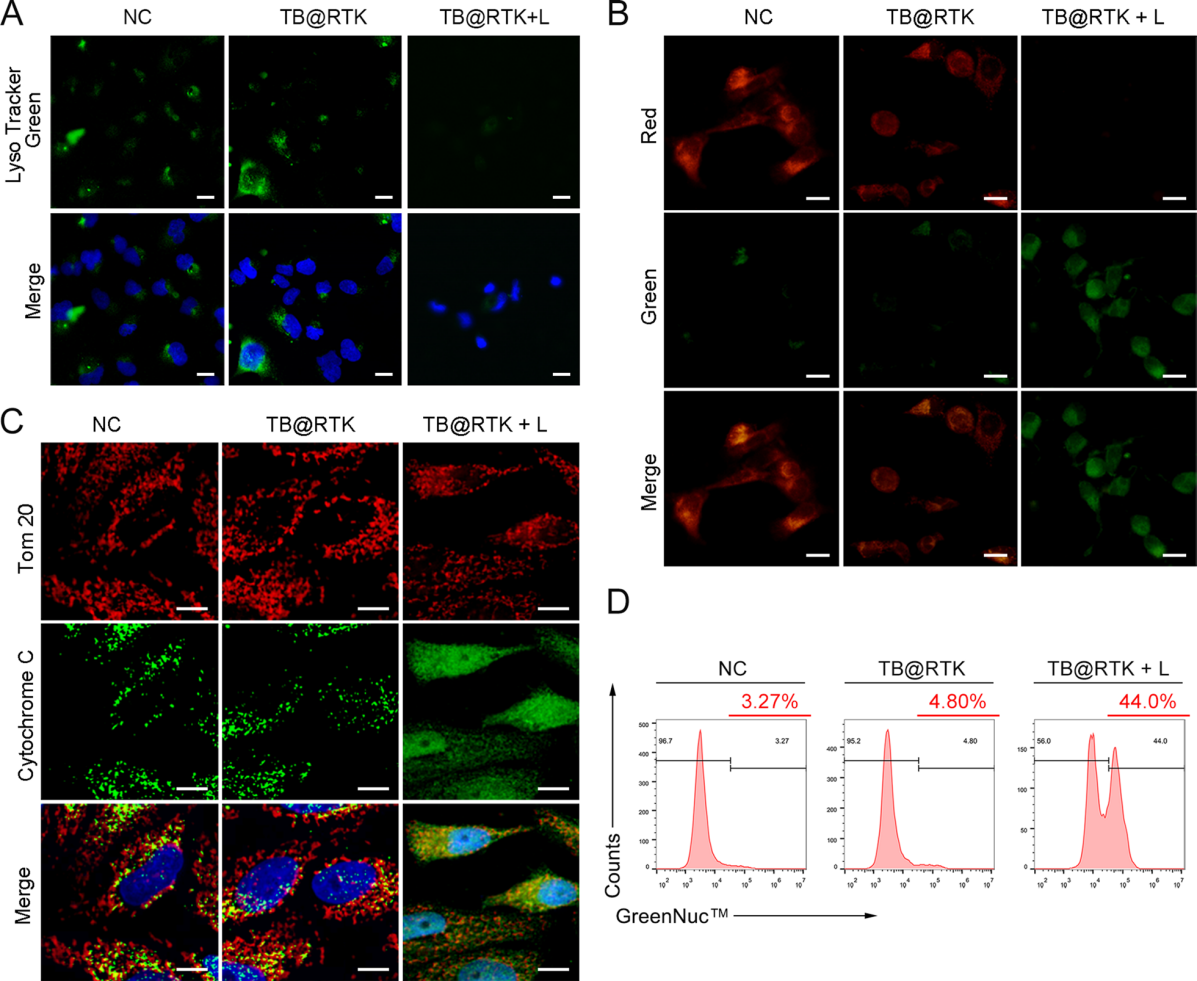
Lysosome disruption, mitochondrial membrane potential depolarization, cytochrome-c release, as well as caspase-3 activation, following TB@RTK micelle-mediated PDT in Hep 1–6 cells. **A** Lysosome disruption was assessed by LysoTracker Green staining after different treatments. In PDT group, the fluorescent intensity decreased. Scale bar: 50 μm. **B** Mitochondrial membrane potential (ΔΨ_m_) in Hep 1–6 cells after the indicated treatments. Scale bar: 20 μm. **C** Immunofluorescence co-staining of cytochrome-c and Tom20. Tom20 indicates the mitochondria. Scale bar: 10 μm. **D** Caspase-3 activation in Hep 1–6 cells after different treatments

ROS generation and lysosome disruption, leading to a release of large amounts of cathepsin from the lysosomes, serve as the primary event that induces mitochondria mediated apoptosis [58]. As shown in Fig. 7A, micelle-mediated PDT could disrupt the integrity of Hep 1–6 cell lysosomes, as observed from an increased lysosomal membrane permeability (Additional file 1: Figure S23), and lysosomal deacidification (Fig. 7A, Additional file 1: Fig. S24). These data suggested that TB@RTK micelle-mediated PDT could trigger a rapid lysosome disruption. Previous studies been elucidated that the release of cytochrome-c from mitochondria due to the disruption of membrane integrity induced by cathepsin is the second event characterizing the mitochondria-mediated apoptotic pathway [58]. We thus detected the mitochondrial membrane potential of Hep 1–6 cells after different treatments by JC-1 fluorescent probe. As shown in Fig. 7B, the mitochondrial membrane potential was significantly damaged in the PDT group. In addition, the release of cytochrome-c from the mitochondria to cytosol was observed after the PDT process, which further confirmed the lose of integrity of mitochondrial (Fig. 7C, Additional file 1: Fig. S25). Once the integrity of the mitochondria was lost, the ATP in the mitochondria was released and secreted outside the tumor cell (Fig. 6B). It has been elucidated that the activation of caspase-3, induced by cytochrome-c released by mitochondria, is the third event resulting in the mitochondria mediated apoptosis [58]. GreenNuc™ Caspase-3 Assay Kit was performed to monitor the activation of caspase-3 in response to TB@RTK micelle-mediated PDT using flow cytometry analysis. It was found that the caspase-3 activity in Hep 1–6 cells treated with TB@RTK micelles with light was elevated most significantly compared to the other groups (Fig. 7D). Taken together, these data verified our hypothesis that mitochondrial mediated apoptosis pathways were involved in the ICD induced by TB@RTK micelle-mediated PDT in Hep 1–6 cells. Moreover, this finding was also confirmed in the Hep G2 cell (Additional file 1: Figure S26). Based on these above studies, we speculated that TB@RTK mediated PDT produced ROS to disrupt lysosomes and release cathepsin, thus leading to mitochondrial damage. Injured mitochondria activate caspase 3 to induce apoptosis. In addition, it has been reported that PDT could cause ER stress to induce membrane translocation of CRT [20]. The translocation CRT induced by ER stress and the release of ATP induced by mitochondrial destruction may be the important causes of ICD induced by TB@RTK-mediated PDT.

### Effects of Chemo-PDT combined with anti-PD-L1 on tumor recurrence and metastasis and the immunological response

**Fig. 8:**
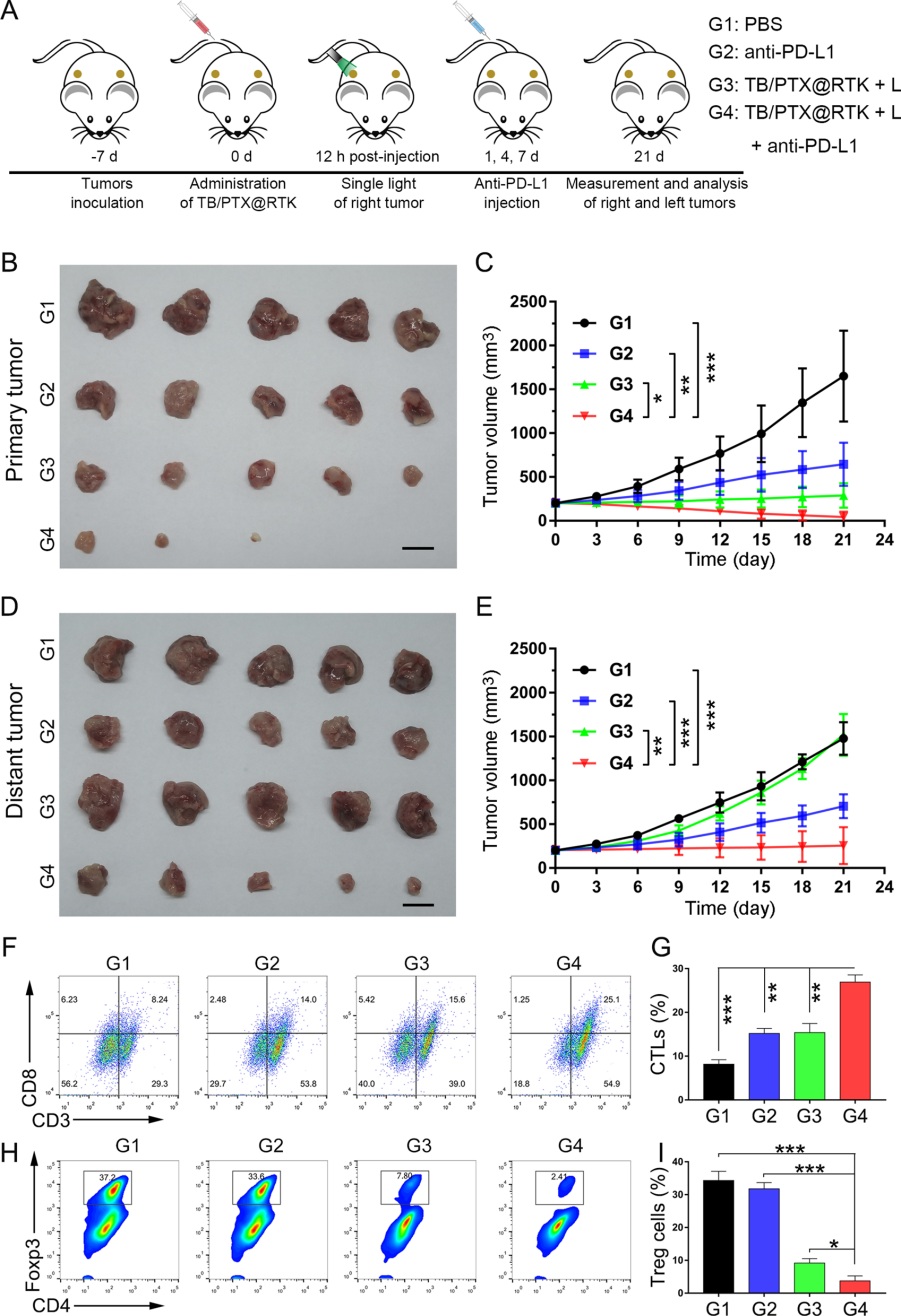
Effects of chemo-PDT combined with anti-PD-L1 (G1, PBS; G2, Anti-PD-L1; G3, TB/PTX@RTK + L; and G4, TB/PTX@RTK + L + anti-PD-L1). **A** Treatment schedule for combination therapy strategy of TB/PTX@RTK micelle-mediated Chemo-PDT and anti-PD-L1. **B**, **C** Primary tumor images and tumor growth curves in the different groups after the indicated treatments. (n = 5). Scale bar: 1 cm. *P < 0.05, **P < 0.01, ***P < 0.001. **D**, **E** Distant tumor images and tumor growth curves in the different groups after the indicated treatments. (n = 5). Scale bar: 1 cm. **P < 0.01, ***P < 0.001. **F**–**I** Representative plots of flow cytometric analysis and statistical analysis of the infiltration of CTLs (CD45^+^CD3^+^CD8^+^; **F** and **G**) and Treg cells (CD3^+^CD4^+^Foxp3^+^; **H** and **I)** in the distant tumors. (n = 5). Scale bar: 1 cm. *P < 0.05, **P < 0.01, ***P < 0.001

Relapse and metastasis are the most challenges for HCC management. At present, there is no widely accepted therapeutic strategy to reduce these risks. Previous studies have showed that PTX or PDT combined with ICIs could suppress tumor recurrence and metastasis [22, 24, 59]. Thus, we hope to verify the effect of TB/PTX@RTK micelle-mediated chemo-PDT combined with anti-PD-L1 antibodies on recurrent and metastasis of tumor. As shown in Fig. 8A, we established bilateral a subcutaneous Hep 1–6 tumor model to mimic primary and distant tumors, in which light irradiation was given on one side (primary tumor) and no light irradiation was given on the other side (distant tumor). Tumor-bearing mice were divided into four groups (n = 5 per group): (1) PBS; (2) anti-PD-L1; (3) TB/PTX@RTK + light; and (4) TB/PTX@RTK + light + anti-PD-L1. The primary tumors in group 3 and group 4 were irradiated (540 nm, 100 mW/cm^2^, 3 min) 12 h after injection of TB/PTX@RTK micelles, and mouse anti-PD-L1 monoclonal antibodies were administered by i.v. injection on day 1, 4, and 7 at a dose of 2.0 mg/kg. The therapeutic effect of each group was dynamically monitored by measuring tumor volume. In addition, the bilateral tumors were dissected and photographed on day 21 after different treatments. We found that treatment with anti-PD-L1 or TB/PTX@RTK + light showed moderate anticancer efficacy on primary tumors, while the combination of TB/PTX@RTK + light with anti-PD-L1 not only inhibited the growth of the primary tumor with no local recurrence during the observation period, but also significantly inhibited the growth of the distant tumors (Fig. 8B-E), suggesting that the combination of TB/PTX@RTK micelle-mediated chemo-PDT therapy with anti-PD-L1 monoclonal antibodies could synergistically enhance systemic anti-tumor effects.

In order to explore the potential mechanism of the synergistic anti-tumor effects of TB/PTX@RTK + light and anti-PD-L1 monoclonal antibodies, the mice were sacrificed to harvest distant tumor tissues and the infiltrated immune cells were analyzed by flow cytometry. The percentages of CTLs (CD45^+^CD3^+^CD8^+^), immunosuppressive Treg cells (CD3^+^CD4^+^Foxp3^+^) and myeloid-derived suppressor cells (MDSCs, CD45^+^CD11b^+^Gr-1^+^) were shown in Fig. 8F–I and Additional file 1: Fig. S26. It was found that the percentage of CTLs increased most significantly in the TB/PTX@RTK + light + anti-PD-L1 group, while both Treg cells and MDSCs were significantly decreased when compared to the other groups (Fig. 8F–I and Additional file 1: Fig. S27). Moreover, the levels of IFN-γ of distant tumor was determined post treatment. Compared with other groups, combination therapy induced the highest levels of IFN-γ in distant tumors (Additional file 1: Figure S28), suggesting that combination therapy could relieve immune suppression and enhance the tumor‐killing activity of CTLs, which then stimulate anti-tumor immunity and migrated to distant tumors to kill tumor cells.

Immunological memory enables the initiation of a more rapid and effective immune response, and effector memory T-lymphocyte (Tem) plays a significant role in anti-tumor immune memory [9, 16]. Therefore, we further examined whether the combination therapy strategy could inhibit tumor relapse. For these experiments, primary tumors were inoculated subcutaneously into the left flanks of C57BL/6 mice treated with TB/PTX@RTK + light or TB/PTX@RTK + light + anti-PD-L1. On day 28, 3 × 10^6^ Hep 1–6 cells were injected subcutaneously into the contralateral flanks of the treated C57BL/6 mice, and tumor volumes was closely monitored for the next 14 day (Additional file 1: Figure S29). The secondary tumors in TB/PTX@RTK + light treatment group grew rapidly, while no tumors were detected in the combined treatment group (Additional file 1: Figure S29). To examine the mechanism by which immunological memory is induced by the combined treatment, we detected Tem cells in the spleen on day 28 by flow cytometry. We found an obvious increase in Tem cells in mice treated with TB/PTX@RTK + light + anti-PD-L1, compared with TB/PTX@RTK + light group (Additional file 1: Figure S30). Taken together, these results indicated that TB/PTX@RTK-mediated chemo-PDT could significantly inhibit primary tumor growth and elicit a systemic anti-tumor immune response. In combination with anti-PD-L1 treatment, the anti-tumor immune effects of combination therapy were extended to inhibit the growth of metastatic and recurrent tumors.

### Toxicity evaluation of TB/PTX@RTK micelles in vivo

**Fig. 9:**
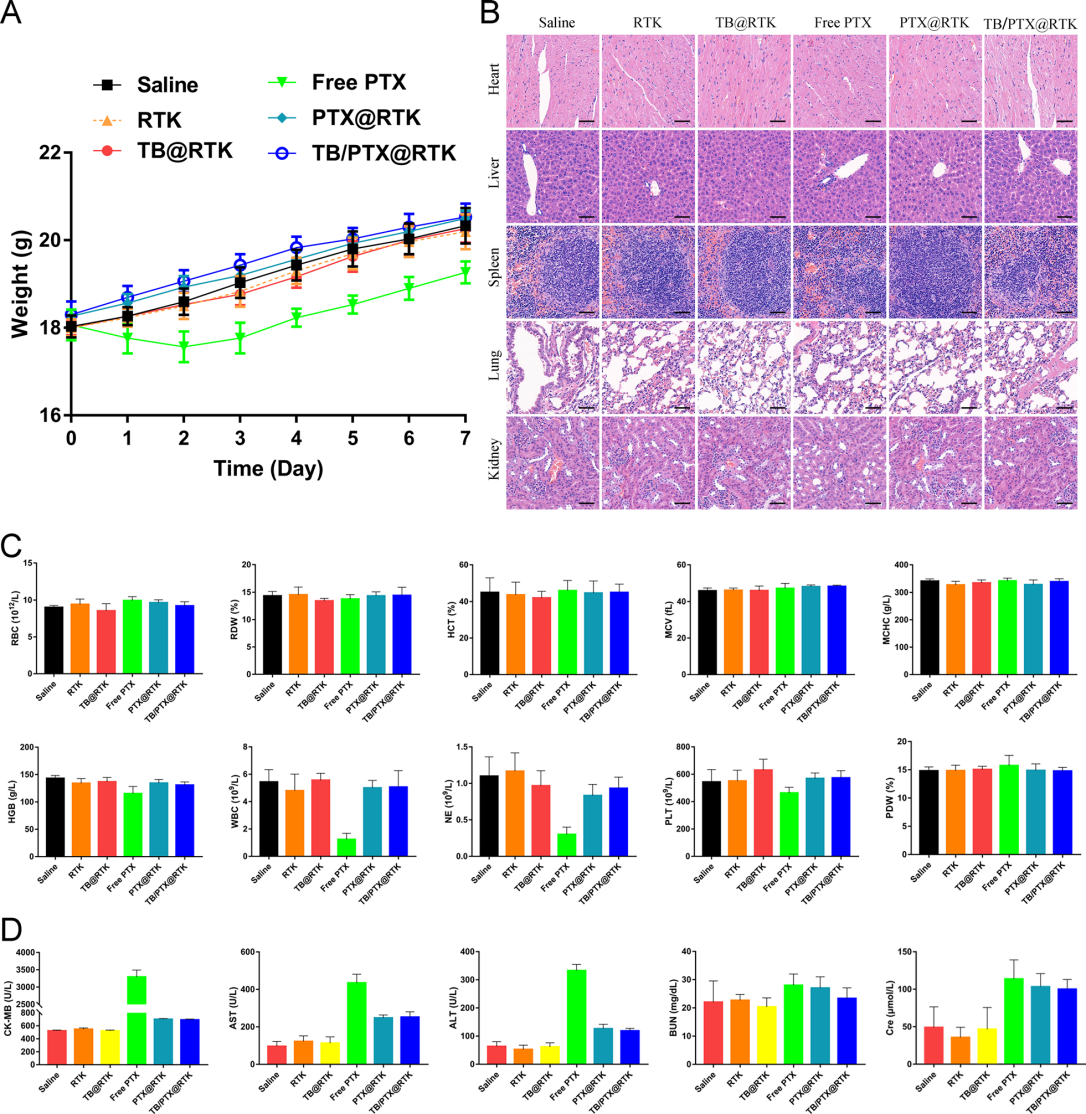
Toxicity of TB/PTX@RTK micelles in vivo. **A** Body weight changes of C57BL/6 mice after injection of saline (negative control), free PTX, and TB/PTX@RTK micelles, respectively. Data represent mean ± SD (n = 3). **B** H&E staining images of various organ slices from C57BL/6 mice 7 days after the indicated treatments. Scale bar: 50 μm. **C** Red blood cells (RBC), red blood cell distribution width (RDW), hematocrit (HCT), mean corpuscular volume (MCV), mean corpuscular hemoglobin concentration (MCHC), hemoglobin (HGB), wight blood cells (WBC), neutrophils (NE), platelet (PLT), and platelet distribution width (PDW) levels in C57BL/6 mice of different groups. **D** The serum levels of CK-MB, ALT, AST, BUN, and Cre in C57BL/6 mice of different groups

In clinic, the use of PTX has been limited to some extent because of its side effects. To assess the side effects of TB/PTX@RTK micelles in mice, we monitored the body weight and multiple organ function of C57BL/6 mice 7 days after the injection of different micelles. Double dose of TB/PTX@RTK micelles used for chemo-PDT (10 mg/kg), was intravenously injected into healthy C57BL/6 mice. To evaluate whether micelles can reduce the side effects of chemotherapeutic drugs, we included the free PTX group as a control. It was found that there was no significant weight loss in the TB/PTX@RTK micelles group compared with the saline (negative control) group (Fig. 9A). In contrast, intraperitoneal injection of free PTX led to weight loss in the mice. In addition, no significant toxicity or tissue damage of TB/PTX@RTK micelles was observed in the major organs by H&E staining (Fig. 9B). Except for free PTX group (same dose as TB/PTX@RTK group), all parameters of blood routine test of the other groups were not significantly abnormal (Fig. 9C). In contrast, free PTX led to a decline in white blood cells and platelets, indicating that free PTX had an inhibitory effect on the bone marrow (Fig. 9C). Then, the blood biochemistry tests including serum creatine kinase-MB (CK-MB), alanine aminotransferase (ALT), aspartate aminotransferase (AST), blood urea nitrigen (BUN), and creatinine (Cre) were also performed (Fig. 9D). The blood biochemistry analysis showed that free PTX led to an increase in serum CK-MB, AST and ALT levels, which suggested that TB/PTX@RTK micelles could avoid the toxic side effects of free PTX. Briefly, these studies demonstrated that TB/PTX@RTK micelles exhibited good biocompatibility and could avoid the toxic side effects of free PTX in vivo.

## Conclusion

In conclusion, we have prepared a targeted and ROS-sensitive TB/PTX@RTK micelle to induce two ICD-inducing modalities for chemo-PDT. Under light irradiation, AIE photosensitizer TB in micelles uses oxygen to generate ROS that not only killed tumor cells during PDT, but also rapidly disintegrated micelles to boost intracellular PTX release in tumor cells, conducing to effective tumor growth inhibition. Furthermore, TB/PTX@RTK micelle-mediated chemo-PDT elicited anti-tumor immune response and upregulated the expression of PD-L1 on tumor cell surface, which could effectively synergize with anti-PD-L1 monoclonal antibodies to induce abscopal effect, and establish long-term immunological memory to inhibit tumor relapse and metastasis. Taken together, the combination of TB/PTX@RTK micelle-mediated chemo-PDT with anti-PD-L1 monoclonal antibodies could synergistically enhance systemic anti-tumor effects.

## Materials and methods

Please see Additional file 1.

## Supplementary Information

**Additional file 1.** Additional figures and tables.

## Data Availability

The datasets used and/or analysed during the current study are available from the corresponding author on reasonable request.

## Abbreviations

HCC: Hepatocellular carcinoma
ICB: Immune checkpoint blockade
PD-1: Programmed cell death protein 1
PD-L1: Programmed cell death ligand 1
TAA: Tumor-associated antigen
CTLs: Cytotoxic T lymphocytes
PDT: Photodynamic therapy
ROS: Reactive oxygen species
ICD: Immunogenic cell death
DAMPs: Damage-associated molecular patterns
nano-DDSs: Nanocarriers-based drug delivery systems
AIE: Aggregation induced emission
PTX: Paclitaxel
TK: Thioketal
PLGA: Polylactic acid-glycolic acid
DCs: Dendritic cells
TEM: Transmission electron microscopy
DLC: Drug loading content
DLS: Dynamic light scattering
CLSM: Confocal laser scanning microscopy
RBCs: Red blood cells
H&E: Hematoxylin–eosin
CRT: Calreticulin
HSP70: Heat shock protein 70
ATP: Adenosine triphosphate
HMGB1: High mobility group box 1
ER: Endoplasmic reticulum
BMDCs: Bone marrow-derived DCs
ELISA: Enzyme-linked immunosorbent assay
MDSCs: Myeloid-derived suppressor cells
Tem: Effector memory T-lymphocyte
CK-MB: Creatine kinase-MB
ALT: Alanine aminotransferase
AST: Aspartate aminotransferase
BUN: Blood urea nitrogen
sCre: Creatinine
