# Supplementary material for: Chemo-photodynamic therapy with light-triggered disassembly of theranostic nanoplatform in combination with checkpoint blockade for immunotherapy of hepatocellular carcinoma
Source: J Nanobiotechnology. 2021 Oct 30;19:355. doi: 10.1186/s12951-021-01101-1 (PMC8557521; doi:10.1186/s12951-021-01101-1)
Supplement: Supplementary file 1 — Additional file 1. Additional figures and tables. [file 12951_2021_1101_MOESM1_ESM.docx]

**Additional file**

**For**

**Chemo-photodynamic therapy with light-triggered disassembly of** **theranostic nanoplatform in combination with checkpoint blockade for immunotherapy of hepatocellular carcinoma**

**Materials and Methods**

PTX, PEG (5k)-TK-PLGA (2k), and NHS-PEG-TK-PLGA used in this study were synthesized by and purchased from Xi’an Ruixi Biological Technology Ltd. (Xi’an, China). Aminated cRGD peptide (RK-5, 187531) used in this study was synthesized by and bought from GL Biochem Ltd. (Shanghai, China). 9,10-Anthracenediyl-bis(methylene)dimalonic acid (ABDA) used in this study was bought from Sigma-Aldrich. The AIEgen photosensitizer of TB was synthesized as previously described [1-2]. ^1^H nuclear magnetic resonance (^1^H NMR) spectrum was measured by Bruker AM 400 apparatus. DLS (Nano-ZS ZEN3690 Malvern Instruments) was used to measure the average size of micelles. TEM (JEM-2100 microscope) was used to observe the morphology of micelles. The DLC of the hydrophobic AIE photosensitizer of TB and the chemotherapeutic drug of PTX was measured by high performance liquid chromatography (HPLC, waters 2695) and UV-vis spectrophotometer (UV-2600, SHIMADZU), respectively.

**Synthesis of cRGD-PEG-TK-PLGA Block Copolymer**

The cRGD peptide was coupled with NHS-PEG-TK-PLGA through an amidation reaction, and then through the self-assembly of cRGD-PEG-TK-PLGA and PEG-TK-PLGA, excellent tumor targeting could be obtained on the prepared micelles of cRGD-modified PEG-TK-PLGA (RTK). In a nutshell, NHS-PEG-TK-PLGA in 5 mL of dimethyl formamide (DMF) was mixed into a solution of cRGD peptide (20 mg, 0.033 mmol) in DMF. After 24 h reaction at room temperature, the product of cRGD-PEG-TK-PLGA was purified by dialysis (MWCO 3500) in ultrapure water for 48 h and then lyophilized. Yield: 74.6%.

**Preparation of the Micelles of RTK, TB@RTK, PTX@RTK, TB/PTX@TK and TB/PTX@RTK**

PEG-TK-PLGA (9 mg), RTK (1 mg), TB (1 mg) and PTX (1 mg) were dissolved in solvent of THF/CHCl_3_ (v/v = 1:1, 1.5 mL) in the dark, ultrapure water (8.5 mL) was then slowly added to that solution. Afterwards, the solution was dialyzed against ultrapure water for 1 d to prepare TB/PTX@RTK micelles with active tumor targeting. In the same way, PEG-TK-PLGA (10 mg), TB (1 mg) and PTX (1 mg) were mixed together to prepare TB/PTX@TK micelles without active tumor targeting. The preparation methods of RTK, TB@RTK and PTX@RTK micelles were similar to that of TB/PTX@RTK micelles.

***In Vitro* Drug Release**

The dialysis method was used to measure the release behavior of PTX from TB/PTX@RTK micelles under different conditions. In a nutshell, 2 mL fresh prepared TB/PTX@RTK micelles solution was transfer into a dialysis bag (MWCO 1000) and then 40 mL of release medium (PBS containing 0.1% Tween 80) was placed into the dialysis bag at 37 °C, accompanied by a steady gentle shake at 100 rpm. At a pre-set time, 2 mL of the release solution was taken out and an equal amount of fresh release medium was added at the same time. PTX concentrations were determined by HPLC. For investigating PTX release behavior, the release medium was contented 10 mM H_2_O_2_ or the micelles were irradiated by light irradiation for 10 min at 100 mW/cm^2^, respectively.

**Cell Lines**

The human HCC cell lines (Hep G2) and mouse HCC cell lines (Hep 1-6) used for testing the imaging and therapeutic efficacy of micelles *in vitro* and *in vivo*, and human normal liver cell lines (L-O2) were purchased from the Cell Bank of the Chinese Academy of Sciences (Shanghai, China). Human kidney proximal tubular cell lines (HK-2) were obtained from American Type Culture Collection. The cells were cultured in RPMI-1640 supplemented with 10% FBS and 1% penicillin–streptomycin (Boster Biological Technology Co. Ltd., CA, USA) at 37 °C in a humidified atmosphere containing 5% CO_2_ [3, 4].

**Experimental Animals**

Male 4~6-week-old Balb/c nude mice and C57BL/6 mice used in this study were provided by Beijing HFK Bioscience Co. Ltd. (Beijing, China) and the Vital River Laboratory Animal Technology Co. Ltd. (Beijing, China). All of the animal experiments and animal feeding were performed in accordance with the guidelines of the Animal Care Committee at Tongji Medical College, HUST, Wuhan, China. This study was performed in compliance with the declaration of Helsinki [3].

**Confocal Imaging**

After being co-incubated with the micelles (5 μg/mL) for 4 h, cells with or without receptor blocking (Cilengitide, 100 nM) were washed twice with PBS for 3 min and then fixed with 4% paraformaldehyde for 35 min. Commercial probe Actin-Tracker Green (Beyotime, Shanghai, China) was used to stain microfilament to show cell contours. Hoechst 33258 (Servicebio, Wuhan, China) was used to localize nuclei. After washing three times with PBS for 5 min, the cell culture slides were transferred to separate object slides and were subjected to confocal imaging analysis directly using the CLSM and relative semi-quantitative fluorescence analysis was performed by using the ImageJ software to assess cellular uptake of micelles.

**Quantitative Analysis of Micelles Uptake by Flow Cytometry**

After being co-incubated with the micelles (5 μg/mL) for 4 h, the cells were washed twice with PBS for 3 min and harvested after digestion with trypsin into flow tubes in the dark. The flow cytometry analysis was performed for relative quantitative analysis of cellular uptake of micelles and cells not co-incubated with micelles were used as the blank control.

**Co-localization between Micelles and Lysosomes**

After being co-incubated with the micelles for 4 h, the cells were washed twice with PBS for 3 min and subsequently incubated with medium supplemented with 75 nM LysoTracker Green (Yeasen Biotech, Shanghai, China) for 2 h at 37 °C. Commercial probe LysoTracker Green was used to localize lysosome. Hoechst 33258 was used to localize nuclei. After washing three times with PBS for 5 min, the cell culture slides were transferred to separate object slides and were subjected to confocal imaging analysis directly using the CLSM and fluorescence co-localization analysis was performed by using the ImageJ software.

**Hemolysis Tests**

In order to perform *in vitro* hemolysis tests, fresh blood (0.5 mL) from orbital venous plexus of C57BL/6 mice was taken into anticoagulant tubes and then diluted with 2 mL PBS. The cell suspension was centrifuged (5000 rpm, 15 min) three times to separate the RBCs from the plasma and the RBCs were then diluted with 5 mL of PBS. 30 μL of the suspension of RBCs was mixed with 170 μL of distilled water (positive control), PBS (negative control), or a solution containing micelles in different concentrations (5~80 μg/mL) into eppendorf tubes. After incubating at room temperature for different pre-set times, the suspension of each tube was centrifuged at 3000 rpm for 15 min. 100 μL of the supernatants of each eppendorf tube was added to a 96-well plate and the Multiskan GO microplate spectrophotometere was used to measure the OD values of supernatants at 570 nm. In addition, the precipitation of each eppendorf tube was used to make the cell smear to observe the morphological changes of erythrocyte [3].

***In Vivo* Toxicity Evaluation of TB/PTX@RTK micelles**

In order to perform *in vivo* blood safety studies of the micelles, 4~6-week-old C57BL/6 mice were divided into six groups (n = 3 per group), and treated with (1) saline (negative control), (2) RTK, (3) TB@RTK, (4) free PTX, (5) PTX@RTK, (6) TB/PTX@RTK at an equal dose of 10 mg/kg via tail vein injection, respectively. We monitored the body weight and multiple organ function of the treated C57BL/6 mice 7 days after different treatment. On the 7th day, the whole blood of mice was collected for hematology analysis immediately [5]. In addition, fresh blood without heparinization from orbital venous plexus of C57BL/6 mice was taken into eppendorf tubes and then kept at room temperature for 30 min to clot and then centrifuged at 5000 rpm for 15 min to separate serum. The serum levels of biochemical markers of major organ function, including CK-MB, ALT, AST, BUN, and sCr, were measured to evaluate the function of major organs, moreover.

***In Vivo* Fluorescence Imaging and Biodistribution Analysis**

HCC tumor models were established to evaluate active tumor targeting of micelles and biodistribution analysis *in vivo*. In a nutshell, 5 × 10^6^ Hep 1-6 cells were inoculated subcutaneously into the Balb/c nude mice to establish HCC tumor models. When the HCC tumor volume reached a mean size of approximately 300 mm^3^, the Balb/c nude mice were intravenously injected with micelles at a concentration of 5 mg/kg based on TB via the tail vein. For the receptor blocking test, mice were intra-peritonelly injected with cilengitide (250 μg) 2 h before injection of the TB/PTX@RTK micelles. After the anaesthesia, the fluorescence imaging of each mouse at 620~720 nm was performed by using an In-Vivo FX PRO at different pre-set times. Subsequently, the Balb/c nude mice were sacrificed to harvest tumors and major organs in the dark, and the fluorescence intensity of tumors and major organs at 620~720 nm was measured by using the same equipment as above.

**Pharmacokinetics Studies of Micelles**

For pharmacokinetics studies, the TB/PTX@RTK micelles or free TB were intravenous injected with C57BL/6 mice via the tail vein. Fresh blood (100 μL) from orbital venous plexus of C57BL/6 mice was taken into anticoagulant tubes at different pre-set times and solubilized with lysis buffer (RIPA) for 30 min, and then subjected to ultrasound for 1 min to fully lyse the blood cells. The fluorescence intensity of the solution at 620~720 nm was measured by using a microplate reader and the exact fluorescence intensity of TB was calculated by deducting that of the blank control. Thereafter, the non-compartment elimination model was used to analyze the data of the relevant pharmacokinetic parameters [6]. The elimination half-life (t1/2) of the TB/PTX@RTK micelles or free TB was calculated by using the WinNonlin v.5.1 software [3].

**Intracellular ROS Detection**

Commercial probe DCFH-DA (Beyotime, Shanghai, China) was used to evaluate intracellular ROS generation by TB-mediated PDT as previously described [3]. After being co-incubated with the micelles for 4 h, the cells were washed three times with PBS for 3 min and then irradiated with a laser irradiation (540 nm, 100 mW/cm^2^, 3 min). After washing twice with PBS for 3 min, Hep G2 and Hep 1-6 cells were subsequently incubated with 1 μM DCFH-DA at room temperature for 30 min in the dark and then captured by using a fluorescence microscope.

**Cytotoxicity Study**

The CCK-8 assays were used to measure Hep G2 and Hep 1-6 cells viability after different treatments as previously described [3]. Flow cytometry analysis was performed to quantify the proportion of apoptotic Hep G2 and Hep 1-6 cells after different treatments by using the Annexin V-FITC/PI Apoptosis Assay Kit (Boster Biological Technology Co. Ltd., CA, USA). Calcein-AM and PI stain were used to evaluate further TB/PTX@RTK-mediated chemo-PDT effects according to the protocol [4]. After being co-incubated with the micelles for 4 h, Hep G2 and Hep 1-6 cells were washed three times with PBS for 3 min and then irradiated with a laser irradiation (540 nm, 100 mW/cm^2^, 3 min) and subsequently incubated Calcein-AM (2 μM) and PI (5 μM) at 37 °C for 20 min, and images were captured with a fluorescence microscope.

***In Vitro* Caspase-3 Assay**

Flow cytometry analysis was performed to quantify the caspase-3 activity of Hep G2 and Hep 1-6 cells after different treatments by using the GreenNuc^TM^ Caspase-3 Activity Detection Kit for Live Cells (Beyotime, Shanghai, China) in accordance with the manufacturer’s protocol [5]. In a nutshell, after the indicated treatments, Hep G2 and Hep 1-6 cells were washed three times with PBS for 3 min and then harvested after digestion with trypsin into flow tubes in the dark. Subsequently, 200 μL of the suspension of HCC cells in the flow tube were incubated with 5 μM of GreenNuc^TM^ Caspase-3 Substrate in the dark at room temperature for 30 min, and the cell samples were immediately analyzed by flow cytometry.

**Lysosomal Membrane Stability**

Lysosomal membrane stability was tested by using acridine orange (AO, Sigma-Aldrich) [7]. After being co-incubated with the micelles for 4 h, Hep G2 and Hep 1-6 cells were washed three times with PBS for 3 min and then irradiated with a laser irradiation (540 nm, 100 mW/cm2, 3 min) and subsequently incubated with complete medium containing AO (5 μg/mL) for 15 min at 37 °C, and images were captured with a fluorescence microscope.

**LysoTracker Green Staining**

After being co-incubated with the micelles for 4 h, Hep G2 and Hep 1-6 cells were washed three times with PBS for 3 min and then irradiated with a laser irradiation (540 nm, 100 mW/cm2, 3 min) and subsequently incubated with 75 nM LysoTracker Green (Yeasen Biotech, Shanghai, China) for 2 h at 37 °C. LysoTracker Green was used to localize lysosome and Hoechst 33258 was used to localize nuclei, and images were captured with a fluorescence microscope.

**Mitochondrial Membrane Potential Analysis**

The mitochondrial membrane potential (ΔΨ_m_) of HCC cells after different treatments was detected by using the JC-1 ΔΨ_m_ Assay Kit as previously described [3, 8]. Briefly, after being co-incubated with the micelles for 4 h, Hep G2 and Hep 1-6 cells were washed three times with PBS for 3 min and then irradiated with a laser irradiation (540 nm, 100 mW/cm2, 3 min) and subsequently incubated with 500 μL of complete medium and 500 μL of JC-1 dyeing working fluid for 20 min at 37 °C and images were captured with a fluorescence microscope.

**Immunofluorescence Analysis**

Immunofluorescence analyses were performed as previously described [3]. Images were captured with a fluorescence microscope, and relative semi-quantitative fluorescence analysis and fluorescence co-localization analysis were performed using ImageJ software. To analyze the expression level of CRT, PD-L1, and HSP70 on the surface of cell, cells after different treatment were fixed with 4% paraformaldehyde. After washing three times with PBS, the cell samples were sealed with goat serum at room temperature for 30 min. After washing twice with PBS, cells were then incubated with the primary antibodies and FITC-conjugated secondary antibody successively in the dark [4]. Red fluorescent probe DiI (Beyotime, Shanghai, China) and Hoechst 33258 were used to label cell membranes and nuclei, respectively [8].

**Immunoblot Analysis**

Immunoblot analyses were performed as previously described to analyze the expression level of acetylated α-tubulin, Detyrosinated α-tubulin, cytochrome c, Bcl-2, and CRT in HCC cells after different treatment [3].

**ELISA Analysis**

The respective supernatant of cells, the cell extract, tumor tissues, and serum after different treatments were collected for measuring the levels of IFN-γ, IL-12, HMGB1, and APT by using the appropriate ELISA Assay Kits and following the manufactures’ instructions [4, 9]. ATP Assay Kit and Mouse HMGB1 Assay Kits were used to detect the extracellular release of ATP and HMGB1, respectively. IL-12 Assay Kits were used to test the serum levels of IL-12 of tumor-bearing C57BL/6 mice after different treatments and to indirectly evaluate the maturation of DCs *in vivo* [9]. IFN-γ Assay Kits were used to test the IFN-γ levels in tumor tissue lysates of tumor-bearing C57BL/6 mice after different treatments and to evaluate the activation of tumor-infiltrating CTLs *in vivo*.

**BMDC Isolation and BMDC Maturation Assays *In Vitro***

To verify the effects of TB/PTX@RTK-mediated chemo-PDT on the maturation of DCs, we extracted bone marrow cells from the femoral bone marrow of C57BL/6 mice according to previous method and used GM-CSF and IL-4 to differentiate and cultured them into immature bone marrow DCs (BMDCs) [4, 10]. After incubation with the micelles for 4 h, the Hep 1-6 cells were washed three times with PBS for 3 min and then laser irradiated with a laser irradiation (540 nm, 100 mW/cm2, 3 min). After incubation for a further 24 h, the medium supernatant of the tumor cells was collected and co-incubated with DCs for 24 h. Ater incubation with anti-CD11c-FITC, anti-CD86-APC, anti-CD80-PE fluorescent antibodies, the BMDCs were detected with a flow cytometer to evaluate the mature of BMDCs. Mature BMDCs were defined as CD11c^+^CD80^+^CD86^+^.

**Antitumor Efficacy Evaluation in the Hep 1-6 Tumor Models**

To evaluate the antitumor effects of TB/PTX@RTK-mediated chemo-PDT in the Hep 1-6 tumor models, Hep 1-6 tumor-bearing C57BL/6 mice were used. When the HCC tumor volume reached a mean size of approximately 200 mm^3^, tumor-bearing C57BL/6 mice were randomly divided into five groups (n = 18 per group): PBS, TB@RTK, TB@RTK + light, TB/PTX@RTK, and TB/PTX@RTK + light, respectively. Micelles (5 mg/kg) were injected through the tail vein. The tumor sites of tumor-bearing C57BL/6 mice were laser irradiated with a laser irradiation (540 nm, 100 mW/cm2, 3 min) 12 h after the injection of the micelles. After different treatments, tumor volumes and weights were recorded everyday. The formula (tumor volume = tumor length × tumor width × tumor width × 0.5) was used to estimate tumor volume as previously described [4]. After 21 days of treatment, tumor-bearing C57BL/6 mice were photographed. To verify the therapeutic efficacy of the TB/PTX@RTK-mediated chemo-PDT, histological changes, proliferation and apoptosis levels of tumors were analyzed by using H&E staining, immunohistochemical analysis and TUNEL assay, respectively, on day 3 after different treatments (n = 5 per group). On day 21 after different treatment, tumor tissues were collected and weighed (n = 5 per group), and the representative tumor tissues were imaged. In addition, the survival time of the rest of tumor-bearing C57BL/6 mice (n = 8 per group) were recorded each day until the last mouse died.

To further evaluate the effects of the combined treatment on primary and metastatic tumors, the bilateral subcutaneous Hep 1-6 tumor models were established. When the HCC tumor volume reached a mean size of approximately 200 mm^3^, tumor-bearing C57BL/6 mice were randomly divided into four groups (n = 5 per group): (1) PBS; (2) anti-PD-L1; (3) TB/PTX@RTK + light; and (4) TB/PTX@RTK + light + anti-PD-L1, respectively. Light irradiation (540 nm, 100 mW/cm2, 3 min) for group 3 and 4 was only given on one side (primary tumor) and no light irradiation was given on the other side (distant tumor) after 12 h postinjection, and anti-PD-L1 was administered by i.v. injection 1, 4, and 7 day at a dose of 2.0 mg/kg. The volume changes of bilateral tumors were detected, and bilateral tumors were dissected and photographed on day 21 after diffferent treatment. In order to clarify the underlying mechanism of the synergistic antitumor effects of TB/PTX@RTK-mediated chemo-PDT and anti-PD-L1 treatment, tumor-bearing C57BL/6 mice were sacrificed to harvest the tumor tissues and the infiltrative immune cells in the distant tumors were analyzed by flow cytometry. The percentages of tumor-killing CTLs (CD45^+^CD3^+^CD8^+^), immunosuppressive Treg cells (CD3^+^CD4^+^Foxp3^+^) and MDSCs (CD45^+^CD11b^+^Gr-1^+^) were analyzed by flow cytometry.

To evaluate effects of combined treatment on tumor recurrence, we rechallenged mice previously treated with TB/PTX@RTK + light or TB/PTX@RTK + light + anti-PD-L1. The primary tumors was inoculated into the left flanks of the C57BL/6 mice as above. When the HCC tumor volume reached a mean size of approximately 200 mm^3^, tumor-bearing C57BL/6 mice were divided into two groups (n = 5 per group) and treated as above. On the 28th day after different treatments, secondary Hep 1-6 tumors were inoculated by injecting 3×10^6^ cells into the right flanks of the mice. The percentage of second tumor-free C57BL/6 mice of the mice in each group (n = 5 per group) were recorded everyday. To investigate the effects of the combined therapy on antitumor immunological memory, splenic effector memory T cells (CD3^+^CD8^+^CD44^+^CD62L^-^) were analyzed by flow cytometry 28 d after treatment.

***In Vivo* Tumor Immune Microenvironment Assays**

To study the effects of cascade chemo-PDT and anti-PD-L1 treatment on the tumor immune microenvironment in vivo, Hep 1-6 subcutaneous tumor model in C57BL/6 mice was used. Tumor tissues and spleen were collected after different treatments and digested to form a single-cell suspension. After lysing the red blood cells, the single-cell suspension was analyzed by flow cytometry. CTLs, Treg cells, MDSCs, and Tem cells were defined as above.

**H&E** **Staining**

H&E staining was performed to assess histological changes of tumors after different treatments as previously described and images were captured using optical microscopy [3].

**TUNEL Assay**

The TUNEL assay was performed to assess apoptosis levels of tumors after different treatments as previously described and images were captured by a fluorescence microscope [3].

**Immunohistochemical Analysis**

Immunohistochemical staining was performed as previously described to analyze the expression level of PD-L1 and Ki-67 in tumor tissue after different treatments and images were captured using optical microscopy [3].

**Statistical Analysis**

All of the measurement data were presented as mean ± standard deviation and were analyzed by utilizing Student’s t-test or the Mann-Whitney U test wherever appropriate. Survival analyses for OS and tumor free survival were performed by utilizing the Kaplan–Meier method and log-rank test. Statistical differences were performed in Graphpad Prism 7.0. *P* < 0.05 was considered statistically significant (**P* < 0.05, ***P* < 0.01, ****P* < 0.001).

**References**

[1] Zhen S, Wang S, Li S, Luo W, Gao M, Ng LG, et al. Efficient Red/Near-Infrared Fluorophores Based on Benzo[1,2-b:4,5-b']Dithiophene 1,1,5,5-Tetraoxide for Targeted Photodynamic Therapy and in Vivo Two-Photon Fluorescence Bioimaging. Adv Funct Mater. 2018; 28: 1706945.

[2] Yi X, Hu J-J, Dai J, Lou X, Zhao Z, Xia F, et al. Self-Guiding Polymeric Prodrug Micelles with Two Aggregation-Induced Emission Photosensitizers for Enhanced Chemo-Photodynamic Therapy. ACS Nano. 2021; 15: 3026-37.

[3] Gao Y, Zheng QC, Xu S, Yuan Y, Cheng X, Jiang S, et al. Theranostic Nanodots with Aggregation-Induced Emission Characteristic for Targeted and Image-Guided Photodynamic Therapy of Hepatocellular Carcinoma. Theranostics. 2019; 9: 1264-79.

[4] Zhou T, Liang X, Wang P, Hu Y, Qi Y, Jin Y, et al. A Hepatocellular Carcinoma Targeting Nanostrategy with Hypoxia-Ameliorating and Photothermal Abilities that, Combined with Immunotherapy, Inhibits Metastasis and Recurrence. ACS Nano. 2020; 14: 12679-96.

[5] Lu Y, Song G, He B, Zhang H, Wang X, Zhou D, et al. Strengthened Tumor Photodynamic Therapy Based on a Visible Nanoscale Covalent Organic Polymer Engineered by Microwave Assisted Synthesis. Adv Funct Mater. 2020; 30: 2004834.

[6] Li M, Gao Y, Yuan Y, Wu Y, Song Z, Tang BZ, et al. One-Step Formulation of Targeted Aggregation-Induced Emission Dots for Image-Guided Photodynamic Therapy of Cholangiocarcinoma. ACS Nano. 2017; 11: 3922-32.

[7] Wang F, Gomez-Sintes R, Boya P. Lysosomal membrane permeabilization and cell death. Traffic. 2018; 19: 918-31.

[8] Liu D, Chen B, Mo Y, Wang Z, Qi T, Zhang Q, et al. Redox-Activated Porphyrin-Based Liposome Remote-Loaded with Indoleamine 2,3-Dioxygenase (IDO) Inhibitor for Synergistic Photoimmunotherapy through Induction of Immunogenic Cell Death and Blockage of IDO Pathway. Nano letters. 2019; 19: 6964-76.

[9] Gao L, Zhang C, Gao D, Liu H, Yu X, Lai J, et al. Enhanced Anti-Tumor Efficacy through a Combination of Integrin αvβ6-Targeted Photodynamic Therapy and Immune Checkpoint Inhibition. Theranostics. 2016; 6: 627-37.

[10] Gao A, Chen B, Gao J, Zhou F, Saeed M, Hou B, et al. Sheddable Prodrug Vesicles Combating Adaptive Immune Resistance for Improved Photodynamic Immunotherapy of Cancer. Nano letters. 2020; 20: 353-62.

**Additional Table and Figures**

**Table S1.** Properties of RTK, TB@RTK, PTX@RTK and TB/PTX@RTK micelles.

| Samples | Size (nm) | PDI | DLC (wt%) | | Mass ration  TB : PTX |
| --- | --- | --- | --- | --- | --- |
|  |  |  | TB | PTX |  |
| RTK | 91.39 ± 0.2 | 0.182 ± 0.017 | /^a^ | /^b^ | /^c^ |
| TB@RTK | 100.4 ± 2.0 | 0.213 ± 0.009 | 7.43 | /^b^ | /^c^ |
| PTX@RTK | 112.9 ± 4.5 | 0.223 ± 0.019 | /^a^ | 6.15 | /^c^ |
| TB/PTX@RTK | 121.2 ± 1.2 | 0.219 ± 0.015 | 7.83 | 5.37 | 1.46 |

^a^No TB was encapsulated into both RTK and PTX@RTK micelles.

^b^No PTX was encapsulated into both RTK and TB@RTK micelles.

^c^TB and PTX do not exist in the RTK, TB@RTK and PTX@RTK micelles at the same time. PDI: polydispersity index.


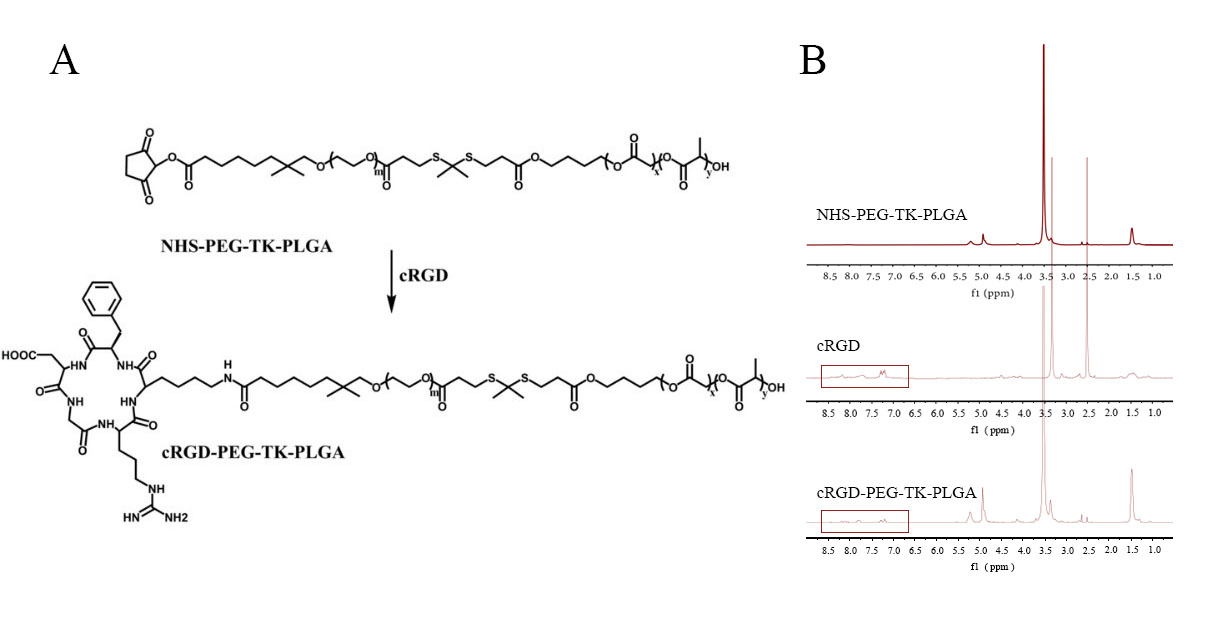


**Figure S1.** (A) Synthesis of ROS-sensitive amphiphilic polymer cRGD-PEG-TK-PLGA. (B) ^1^H NMR spectra (400 MHz, DMSO‐d6) of NHS-PEG-TK-PLGA, cRGD and cRGD-PEG-TK-PLGA.


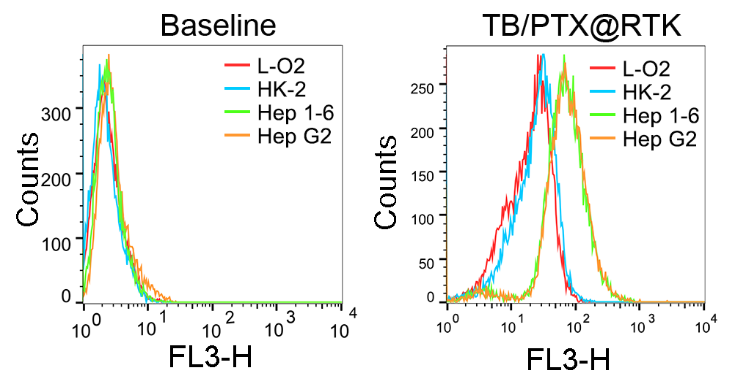


**Figure S2.** Flow cytometric analyses of the fluorescence intensity of L-O2, HK-2, Hep 1-6 and Hep G2 cells after incubation with the TB/PTX@RTK micelles (5 μg/mL) for 4 h.


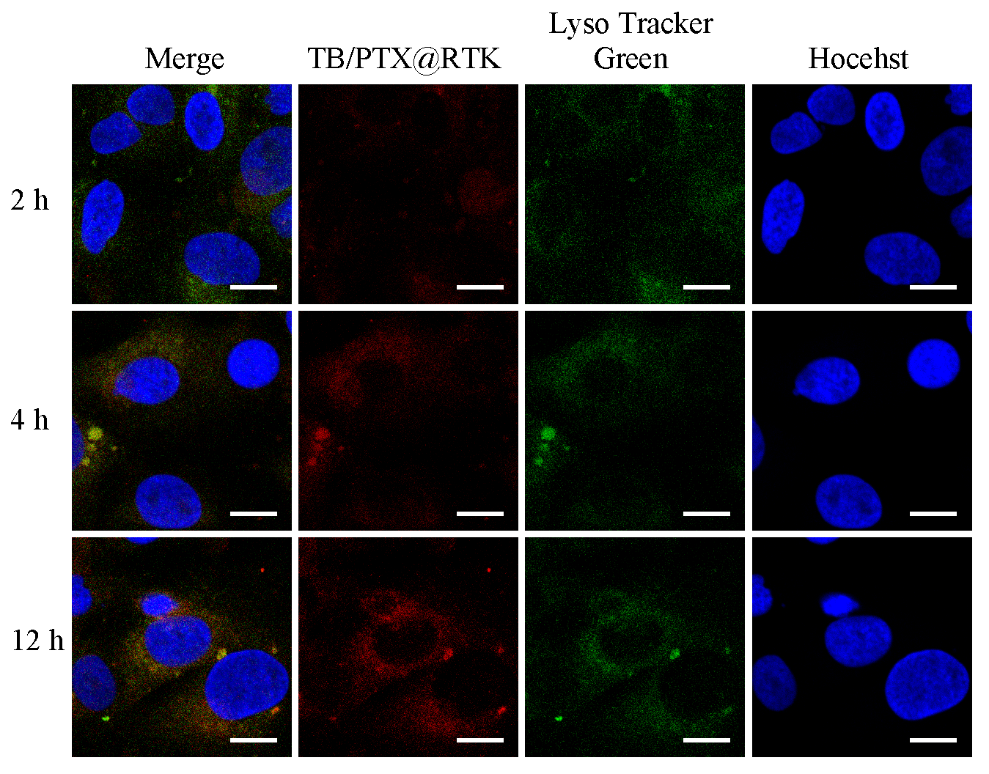


**Figure S3.** The cell uptake and subcellular location of TB/PTX@RTK micelles at different time points in Hep G2 cells. Scale bars: 20 μm.


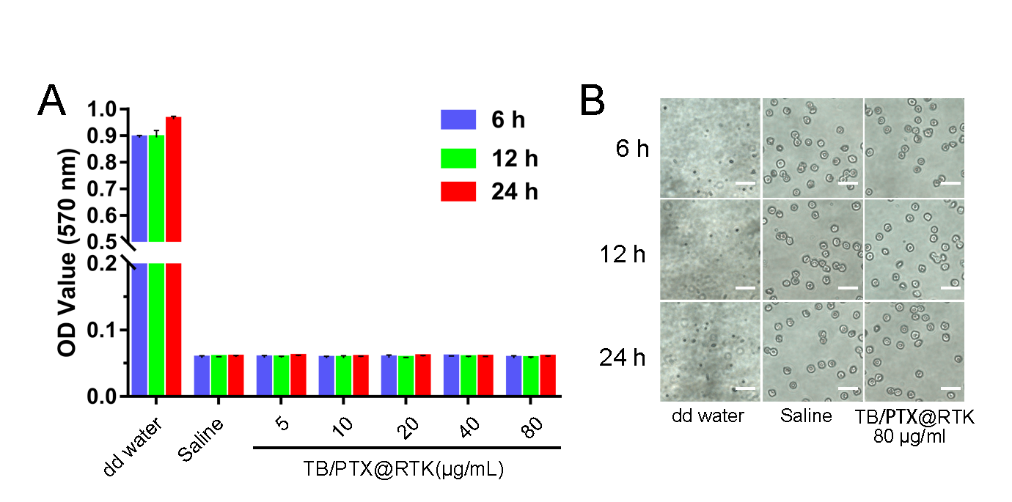


**Figure S4.** Hemocompatibility evaluation of TB/PTX@RTK micelles. (A) Hemolysis of red blood cells in the presence of TB/PTX@RTK micelles with various concentrations at preset time. Data represent mean ± SD (n = 3). (B) Optical microscopic observation of the dispersion states of the erythrocytes after incubation with distilled water, saline, and TB/PTX@RTK micelles for 6, 12, and 24 h, respectively. Scale bars: 20 μm.


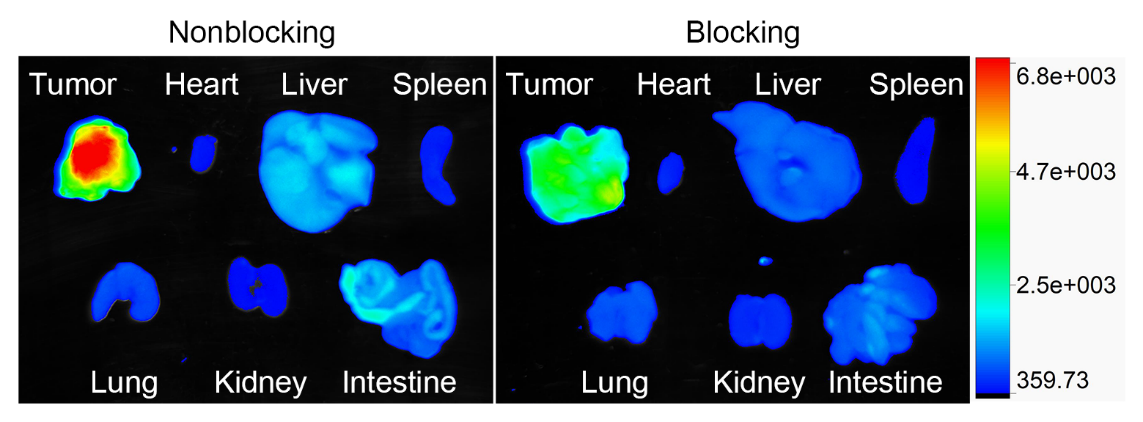


**Figure S5.** *Ex vivo* fluorescence imaging of tumor tissues and various organs from tumor-bearing mice 12 h after intravenous injection with or without blocking the receptors by cilengitide.


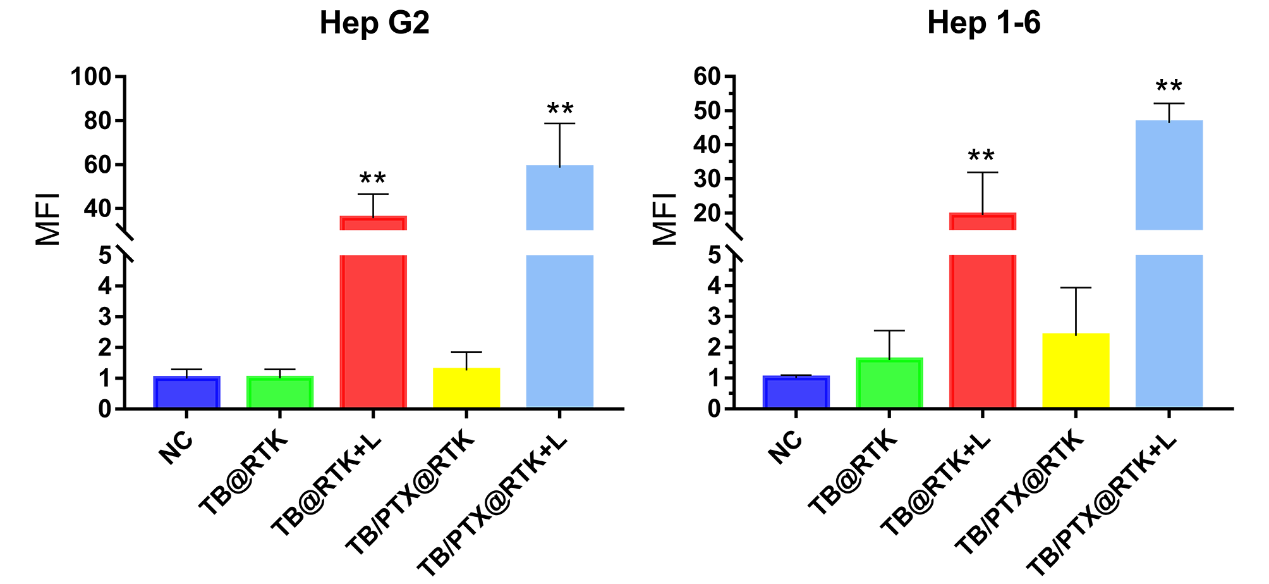


**Figure S6.** Semi-quantitative analysis of fluorescence intensity of ROS production in Hep G2 and Hep 1-6 cells after different treatments. Data represent mean ± SD (n = 3); compared with NC group, ***P* < 0.01.


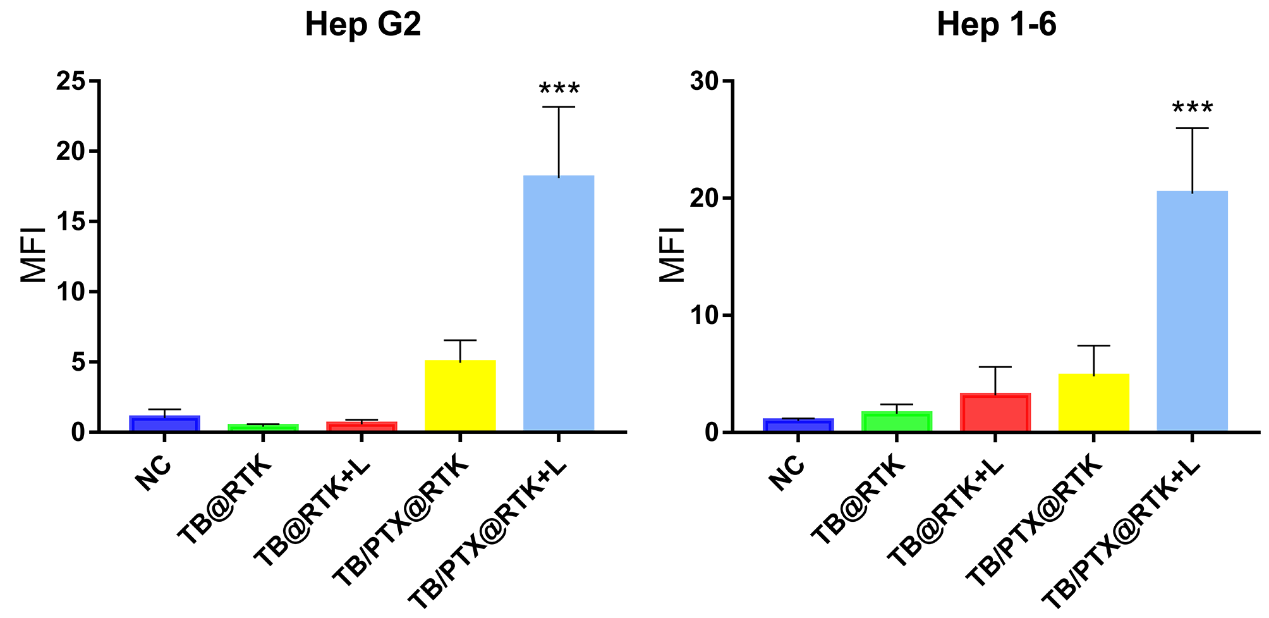


**Figure S7.** Immunofluorescence semi-quantitative analysis of acetylated α-tubulin in Hep G2 and Hep 1-6 cells after different treatments. Data represent mean ± SD (n = 3); compared with NC group, ****P* < 0.001.


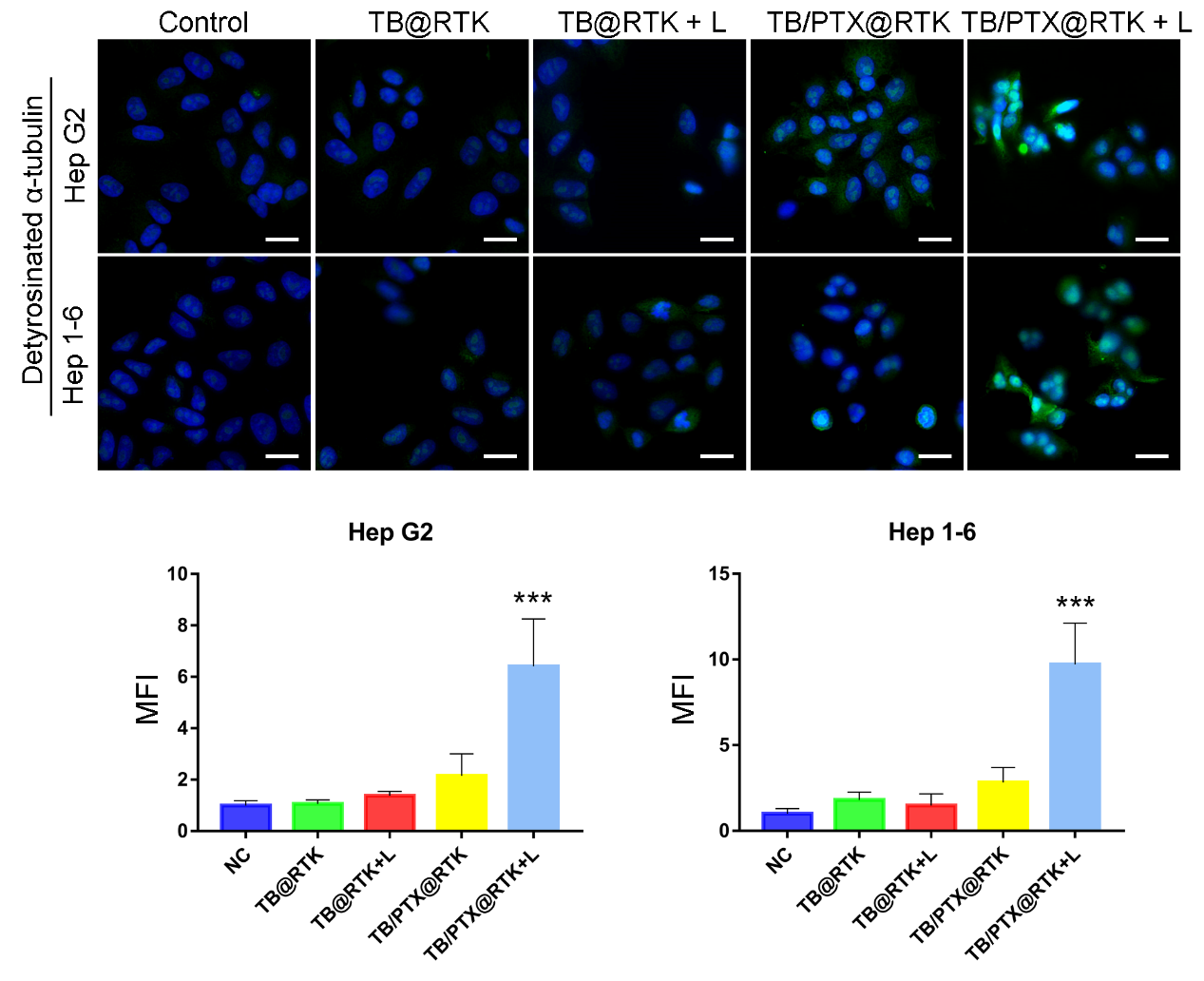


**Figure S8.** Immunofluorescence staining and semi-quantitative analysis of detyrosinated α-tubulin in Hep G2 and Hep 1-6 cells after different treatments. Scale bars: 20 μm. Data represent mean ± SD (n = 3); compared with NC group, ****P* < 0.001.


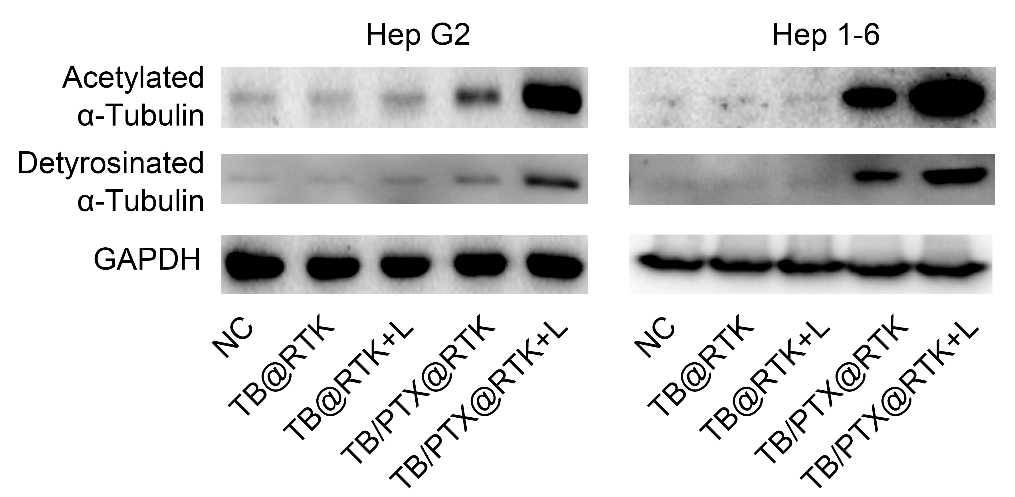


**Figure S9.** Western blot analysis of acetylated α-tubulin and detyrosinated α-tubulin in Hep G2 and Hep 1-6 cells after different treatments.


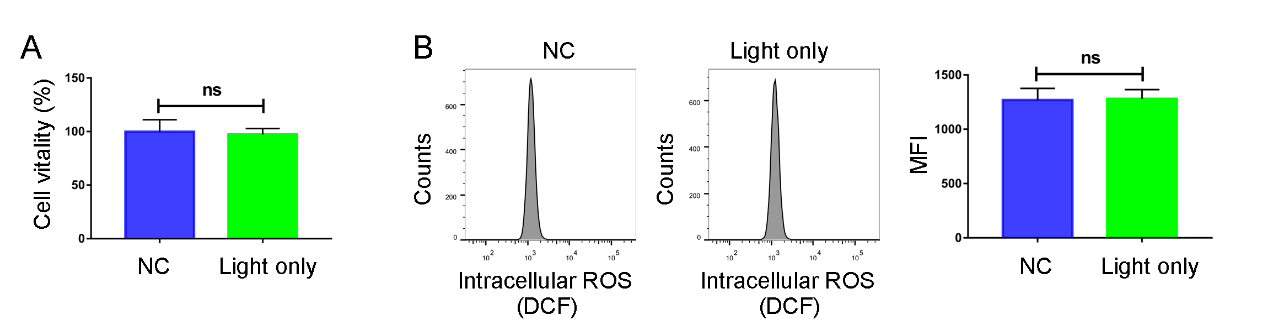


**Figure S10.** Effects of light on cell activity (A) and production of ROS (B) in Hep G2 cells. Ns: no significant difference.


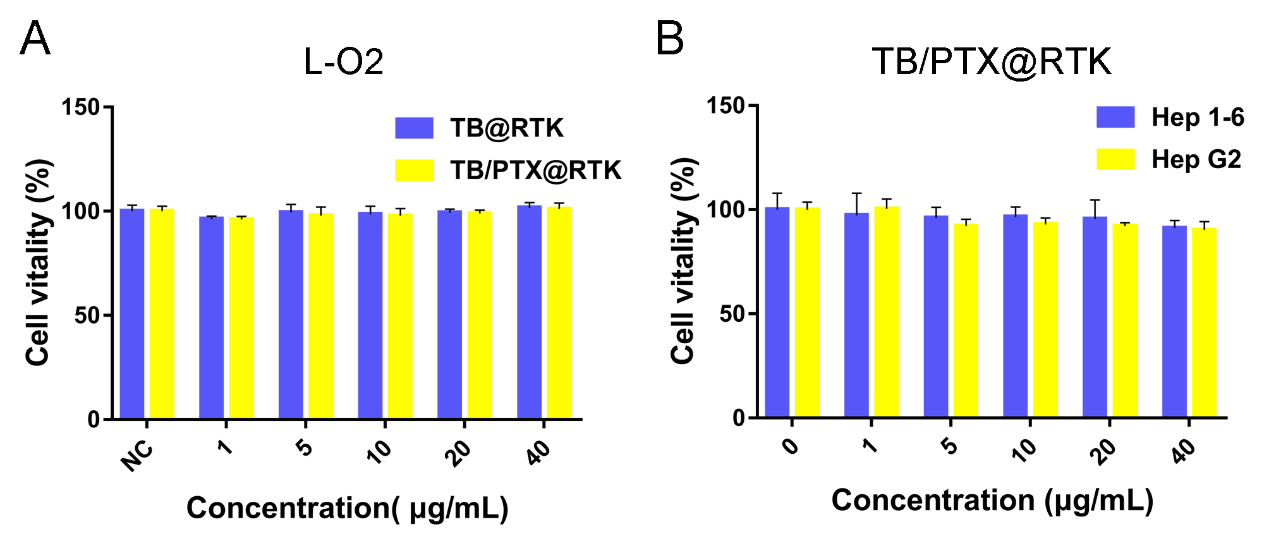


**Figure S11.** Cytotoxicity of micelles in vitro. (A) Cell viability after incubation with 1~40 μg/mL TB@RTK or TBPTX@RTK micelles in L-O2 cells. (B) Cell viability after incubation with 1~40 μg/mL TB/PTX@RTK micelles in Hep 1-6 and Hep G2 cells.


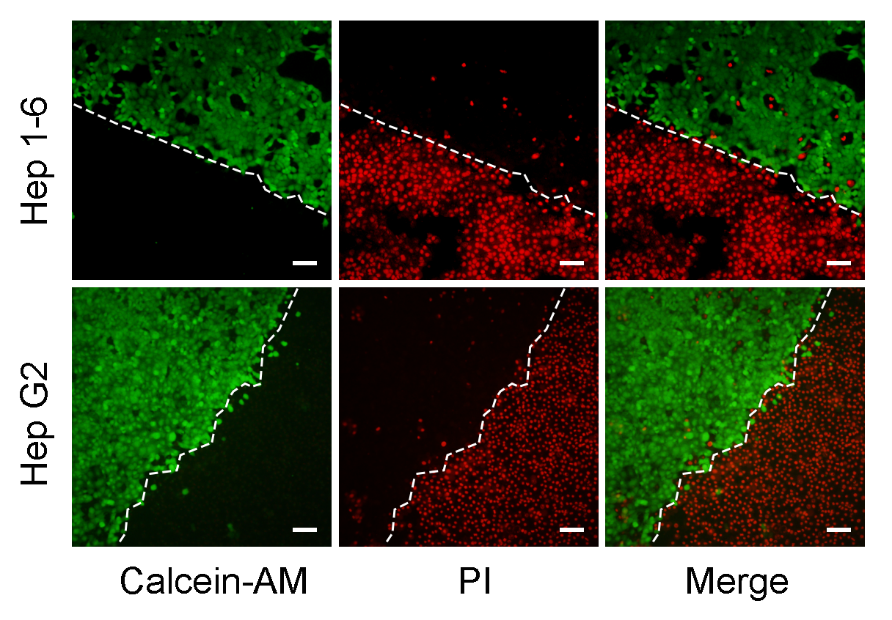


**Figure S12.** Fluorescence images of cells after incubation with TB/PTX@RTK micelles, then with or without irradiation (red and green indicate nonviable and viable cells, respectively). The areas below the white dotted lines were irradiated. Scale bars: 100 μm.


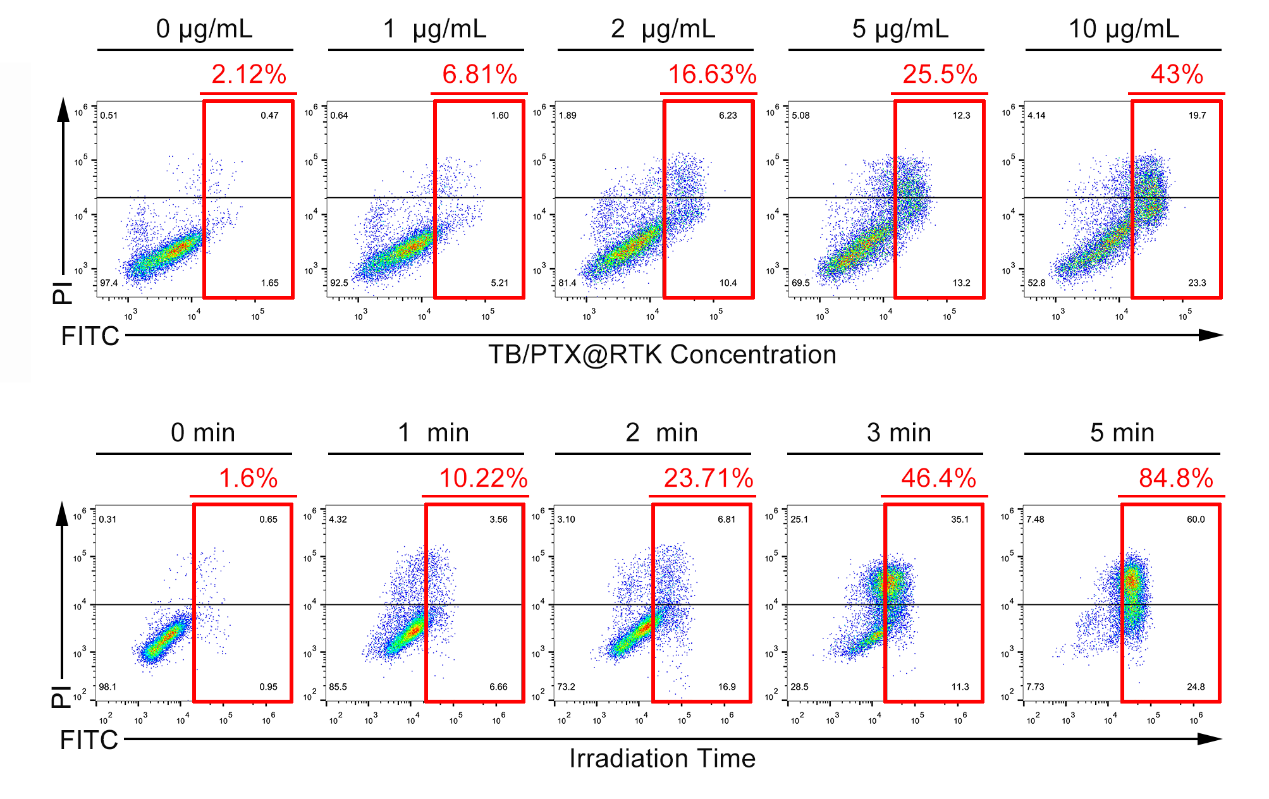


**Figure S13.** Flow cytometry analysis of the effect of increasing TB/PTX@RTK concentration (irradiation 3 min) or irradiation time (TB/PTX@RTK, 5 μg/mL) on cytotoxicity in Hep G2 cells. The right area (Annexin V-FITC positive) indicates apoptotic cells.


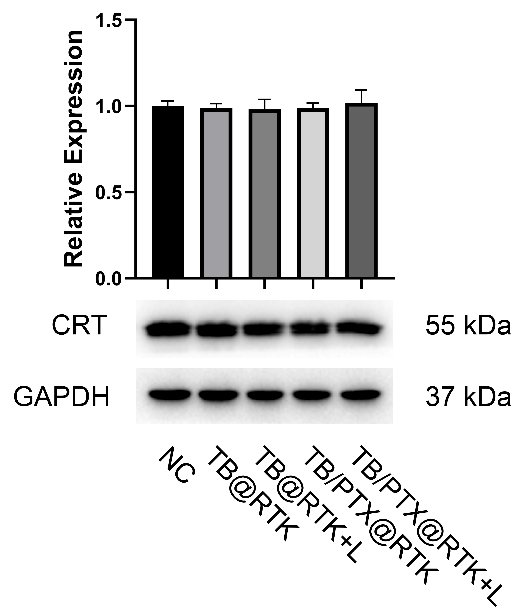


**Figure S14.** Western blot analysis of CRT in Hep 1-6 cells after different treatments. Data represent mean ± SD (n = 3).


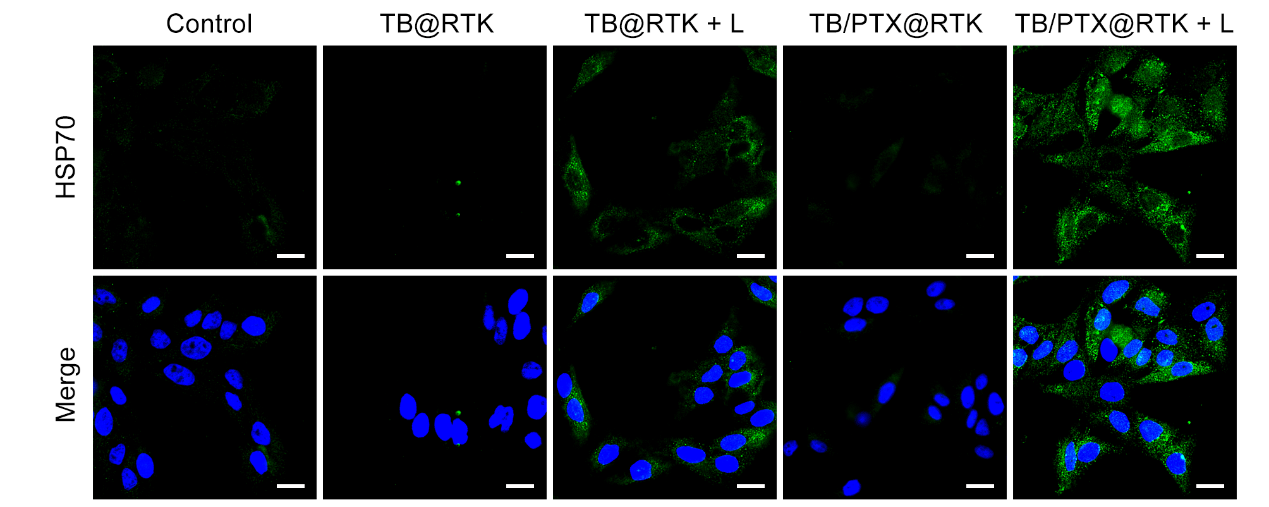


**Figure S15.** Immunofluorescence staining analysis of HSP70 expression on the cell surface after different treatments. Scale bar: 20 μm.


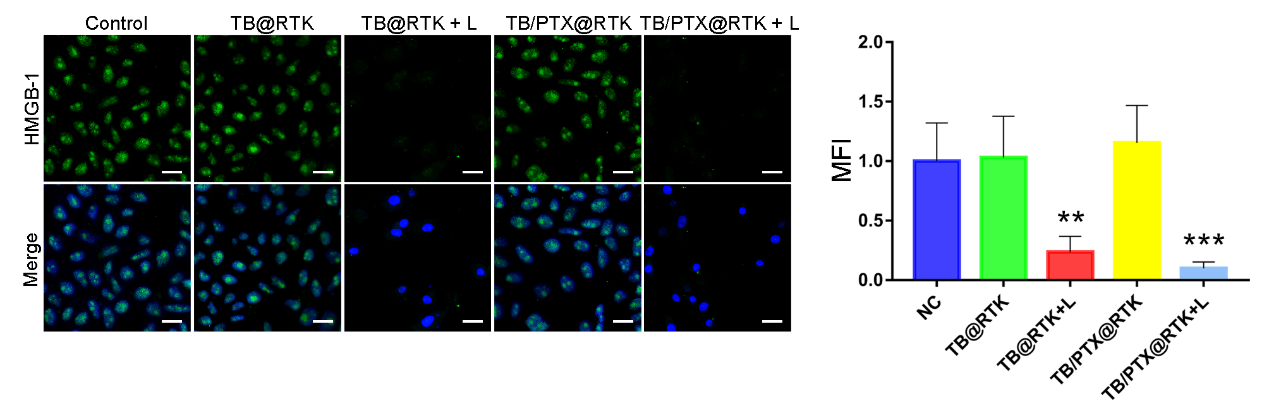


**Figure S16.** Immunofluorescence staining and semi-quantitative analysis of HMGB1 in Hep 1-6 cells after different treatments. Scale bars: 20 μm. Data represent mean ± SD (n = 3); compared with NC group, ***P* < 0.01, ****P* < 0.001.


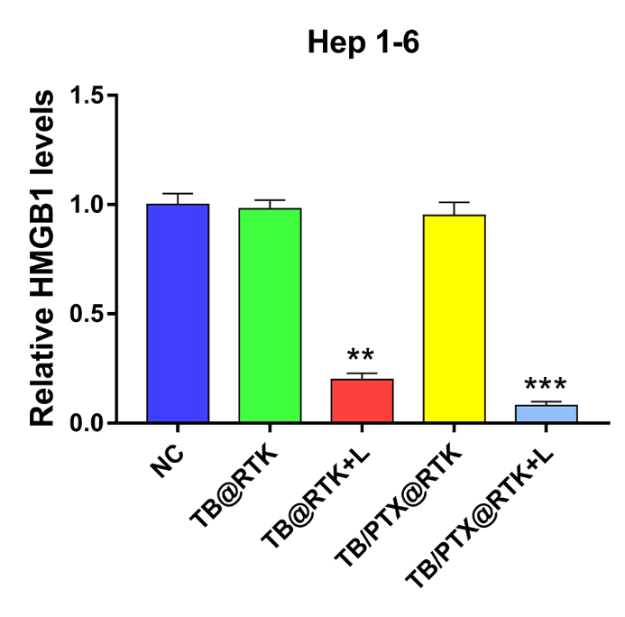


**Figure S17.** Intracellular HMGB1 levels in Hep 1-6 cells after different treatments as measured by Elisa kit. Data represent mean ± SD (n = 3); compared with NC group, ***P* < 0.01, ****P* < 0.001.


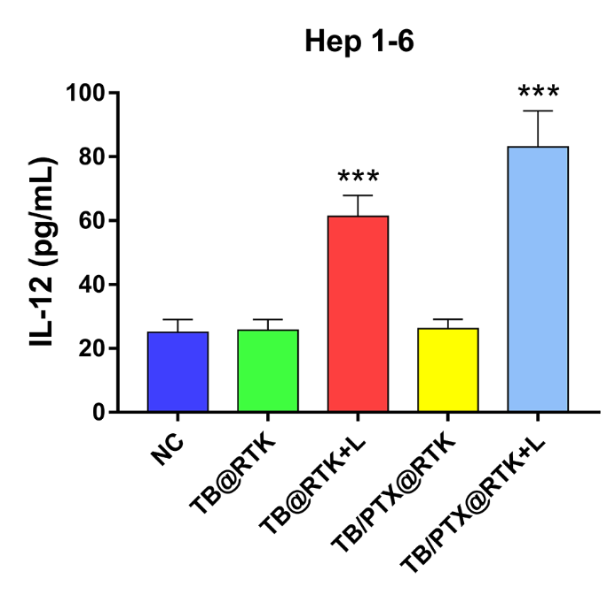


**Figure S18.** IL-12 levels in the serum from tumor-bearing mice after different treatments as measured by Elisa kit. Data represent mean ± SD (n = 3); compared with NC group, ****P* < 0.001.


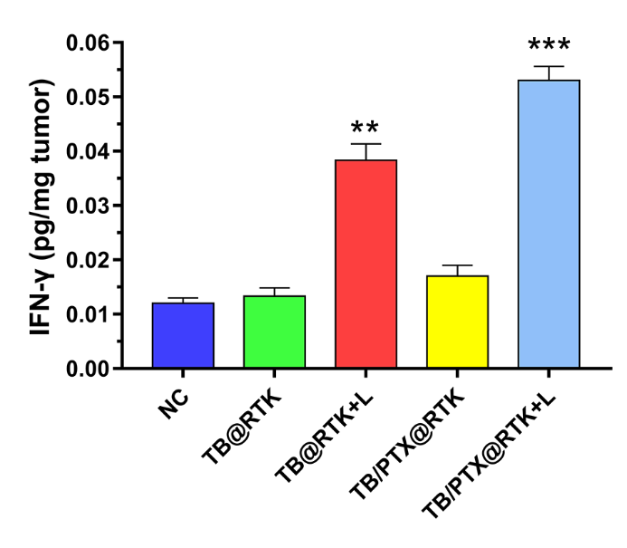


**Figure S19.** Elisa analysis of IFN-γ with respect to tumors after different treatments. Data represent mean ± SD (n = 3); compared with NC group, ***P* < 0.01, ****P* < 0.001.


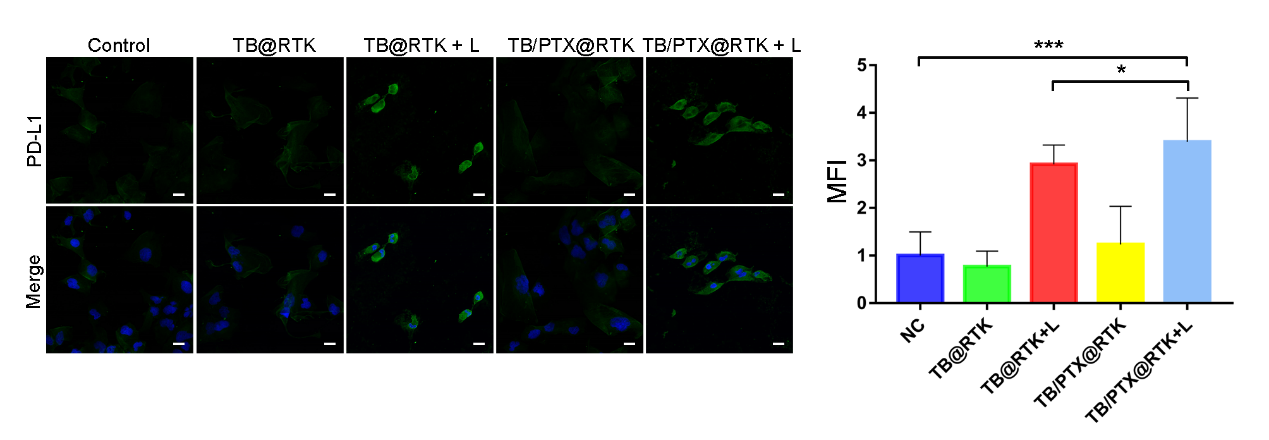


**Figure S20.** Immunofluorescence staining and semi-quantitative analysis of PD-L1 in Hep 1-6 cells after different treatments *in vitro*. Scale bars: 20 μm. Data represent mean ± SD (n = 3); compared with NC group, ***P* < 0.01, ****P* < 0.001.


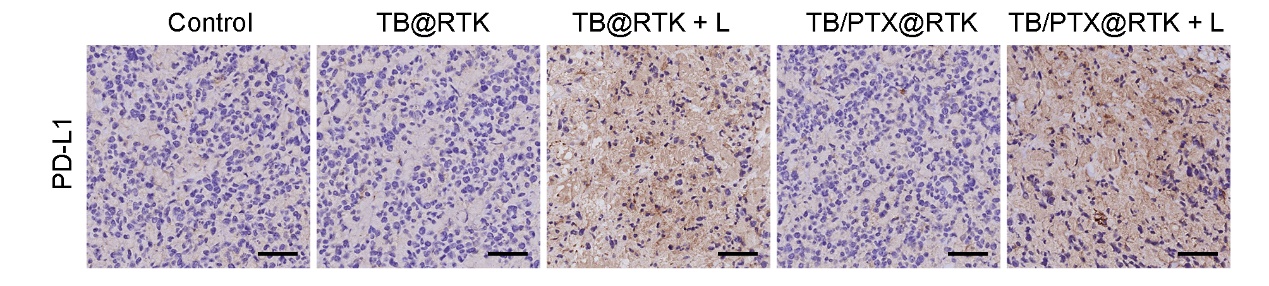


**Figure S21.** Immunohistochemical analysis of PD-L1 in HCC tumor tissues after different treatments in vivo. Scale bars: 50 μm.


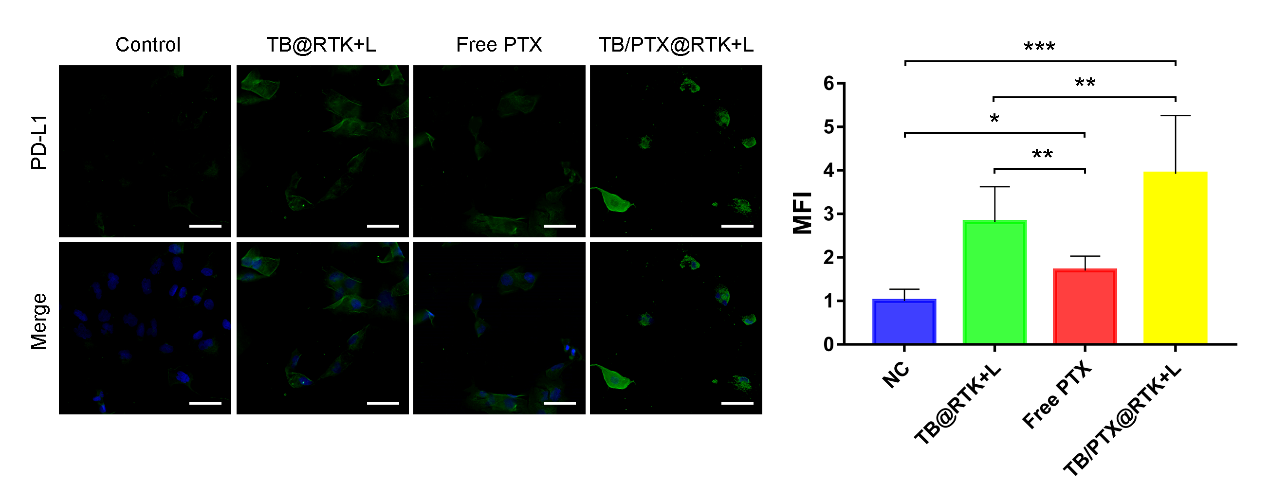


**Figure S22.** Immunofluorescence staining and semi-quantitative analysis of PD-L1 in Hep 1-6 cells after different treatments *in vitro*. Scale bars: 50 μm. Data represent mean ± SD (n = 3); **P* < 0.05, ***P* < 0.01, ****P* < 0.001.


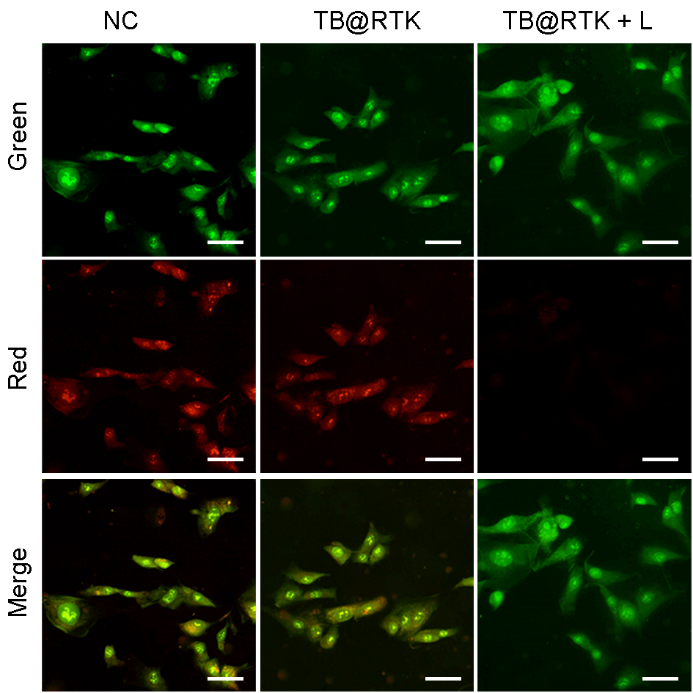


**Figure S23.** Acridine orange staining of Hep 1-6 cells after different treatments. Red fluorescence indicates the lysosome and green fluorescence indicates the nucleic acid. Scale bar: 50 μm.


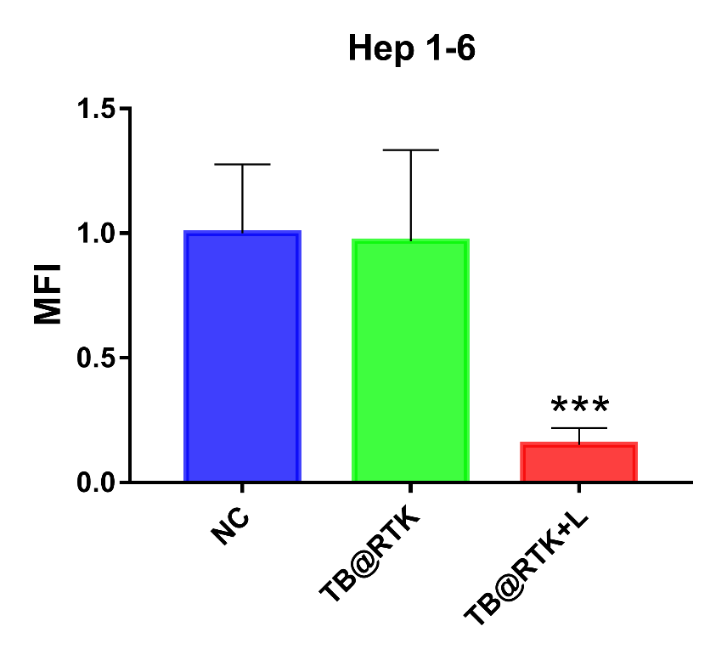


**Figure S24.** Semi-quantitative analysis of LysoTracker Green in Hep 1-6 cells after different treatments. Data represent mean ± SD (n = 3); compared with NC group, ****P* < 0.001.


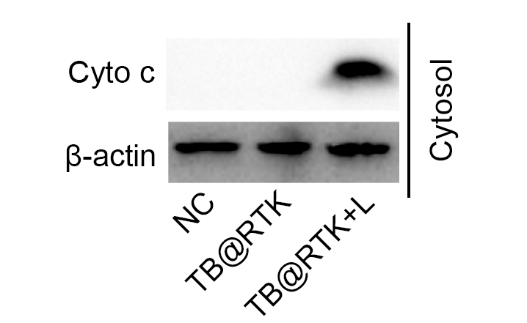


**Figure S25.** Western blot analysis of cytochrome c release in Hep 1-6 cells after different treatments.


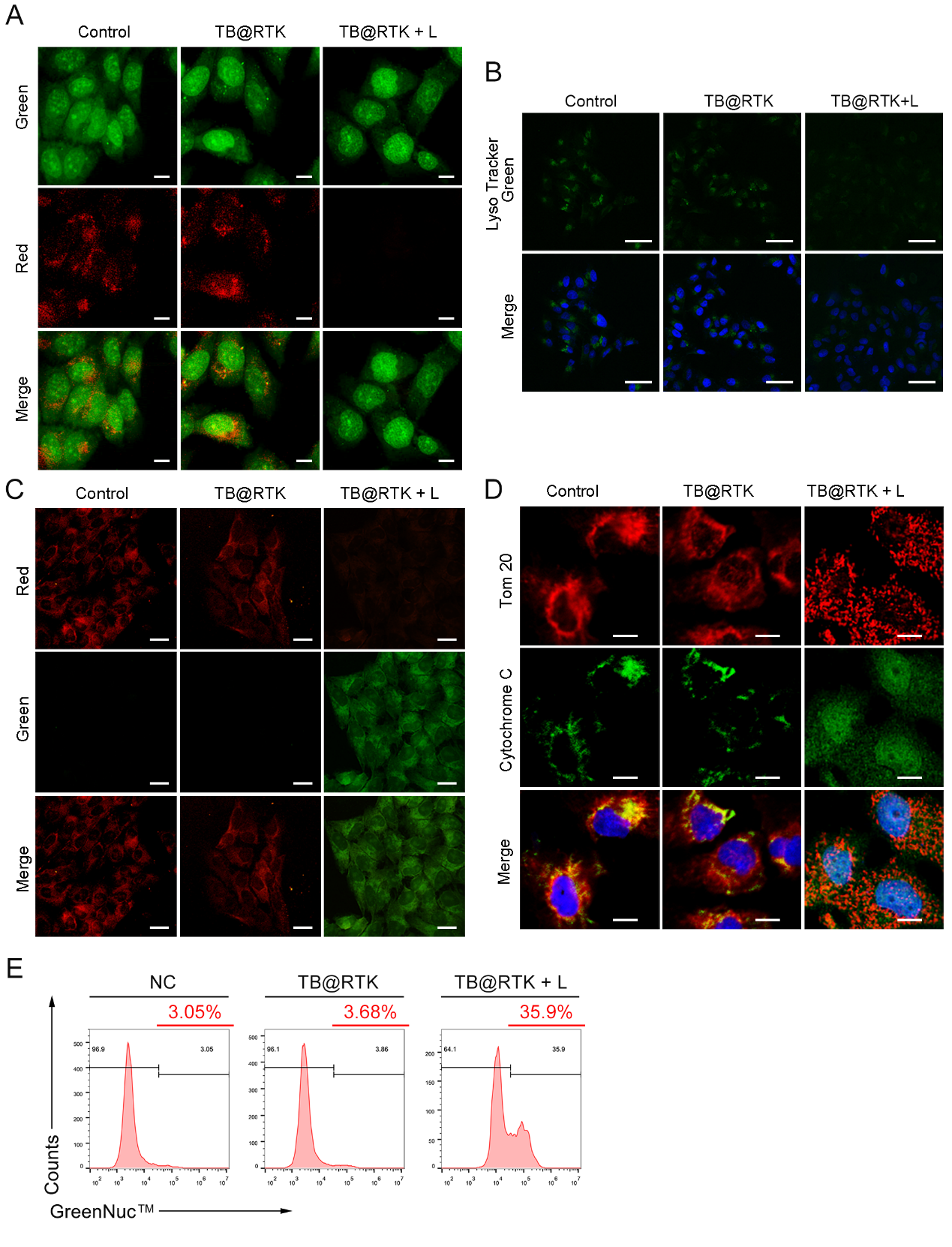


**Figure S26.** Lysosome disruption, mitochondrial membrane potential depolarization, cytochrome-c release, as well as caspase-3 activation, following PDT based on micelles on Hep G2 cells. (A) Acridine orange staining of HCC cell lines Hep G2 cells after different treatments. Red fluorescence indicates the lysosome and green fluorescence indicates the nucleic acid. Scale bar: 10 μm. (B) LysoTracker Green was used to stain lysosome after different treatments. In PDT group, the fluorescent intensity decreased. Scale bar: 50 μm. (C) Effects of micelle-mediated PDT on mitochondrial membrane potential (ΔΨ_m_) in Hep G2 cells after different treatments. Scale bar: 20 μm. (D) Immunofluorescence co-staining of cytochrome-c and Tom20. Tom20 indicates the mitochondria. Scale bar: 10 μm. (E) Caspase-3 activation in Hep G2 cells after different treatments.


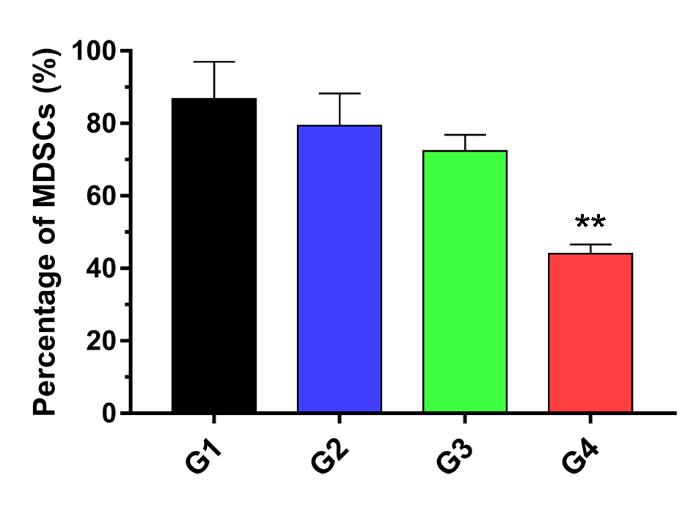


**Figure S27.** The frequency of MDSCs (CD11b^+^Gr-1^+^) in tumor-infiltrating CD45^+^ cells in the distant tumors (G1, PBS; G2, Anti-PD-L1; G3, TB/PTX@RTK + L; and G4, TB/PTX@RTK + L + anti-PD-L1). Data represent mean ± SD (n = 3); compared with NC group, ***P* < 0.01.


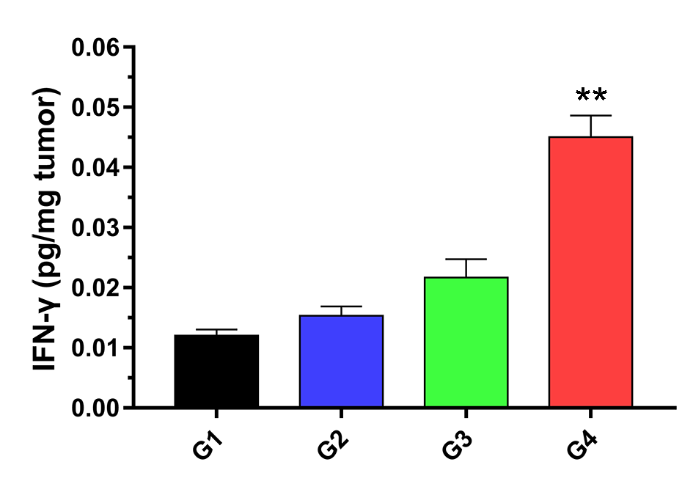


**Figure S28.** Elisa analysis of IFN-γ with respect to distant tumors after different treatments (G1, PBS; G2, Anti-PD-L1; G3, TB/PTX@RTK + L; and G4, TB/PTX@RTK + L + anti-PD-L1). Data represent mean ± SD (n = 3); compared with NC group, ***P* < 0.01.


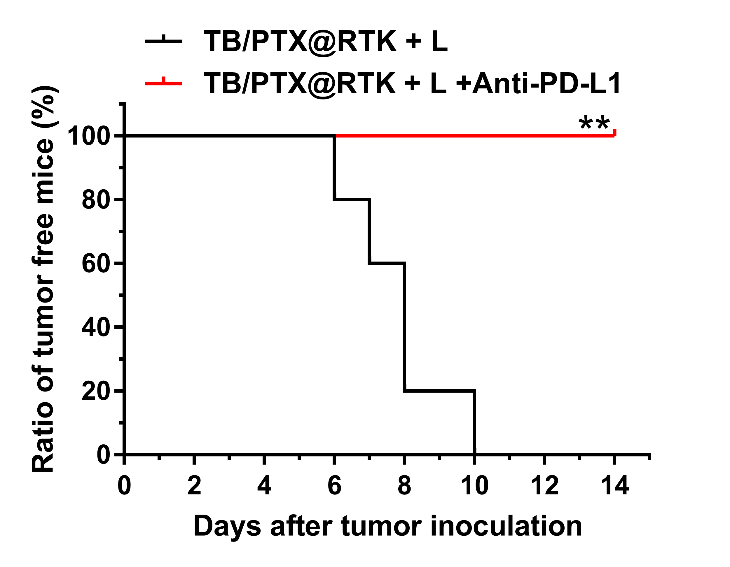


**Figure S29.** Tumor recurrence inhibition. Kaplan-Meier curves showing the percentage of second tumor-free mice after different treatments. ***P* < 0.01.


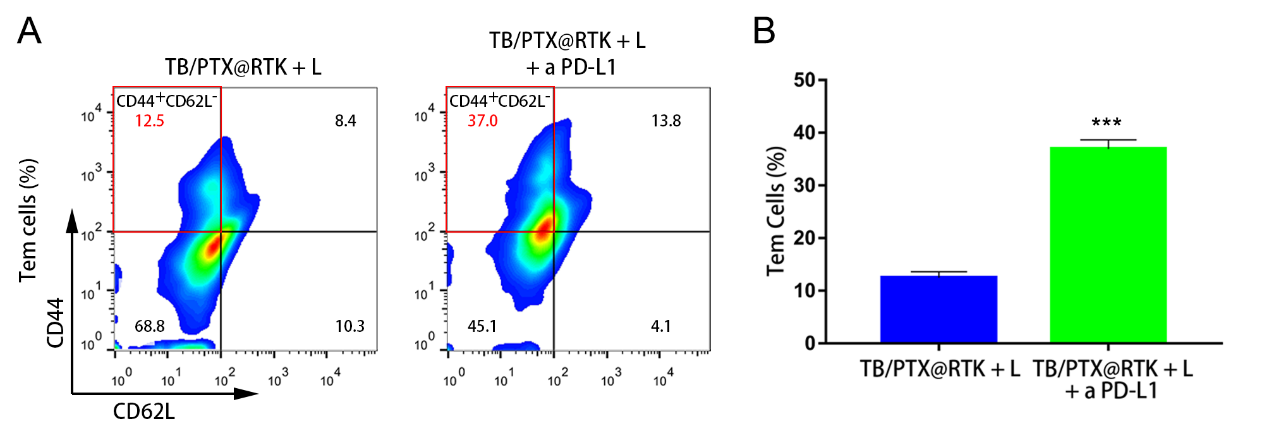


**Figure S30.** Immunological memory effects induced by chemo-PDT combined with anti-PD-L1 therapy. (A) Representative flow cytometric plots of CD3^+^CD8^+^CD44^+^CD62L^-^ Tem cells. (B) Percentage of Tem cells in the spleens of rechallenged mice. ****P* < 0.001.
